# Microwave MIMO E‑Nose for Wireless Communication and Selective Detection of VOC Mixtures with Concentration Estimation

**DOI:** 10.1021/acssensors.5c00243

**Published:** 2025-08-25

**Authors:** Mohammad Mahmudul Hasan, Onur Alev, Pavel Skrabanek, Gabriela Soukupová, Fatima Hassouna, Michael Cheffena

**Affiliations:** † Faculty of Engineering, 8018 Norwegian University of Science and Technology, Gjøvik 2815, Norway; ‡ Department of Physics, Gebze Technical University, 41400, Gebze, Kocaeli, Turkey; § Faculty of Mechanical Engineering, 48274 Brno University of Technology, Brno 61200, Czech Republic; ∥ Faculty of Chemical Engineering, University of Chemistry and Technology, Prague 16628, Czech Republic

**Keywords:** MIMO, E-nose, MIP, CNT, VOC, molecularly imprinted polymer, carbon nanotubes, antenna sensor, microwave sensor, dual-functional, gas sensor, selective, volatile organic compounds, mixture sensor, ethanol, ethanol, isopropanol, butanol, wireless sensor network, neural network, machine learning, decoupling and matching network

## Abstract

We present the first dual-functional microwave electronic nose (E-nose) that enables wireless communication, VOC mixture detection, and reliable concentration estimation, designed for seamless integration with wireless sensor networks. The proposed E-nose features multiple-input multiple-output (MIMO) antenna system functionalized with molecularly imprinted polymer (MIP) and multiwalled carbon nanotube-based sensing materials for the selective detection of individual or mixed volatile organic compounds (VOCs). We addressed several novel challenges such as managing cross-reactivity under electromagnetic interference with wideband decoupling, employing a dual-branch neural network (NN) with feature prioritization and transducer behavior insights, and optimizing sensor placement for spatial isolation in a compact design. The developed E-nose demonstrated high sensitivity and selectivity for VOC mixtures at room temperature, with detection limits below safety thresholds, ensuring practical applicability. The optimized NN model achieved high predictive accuracy (*R* ^2^ = 0.982 to 0.991), delivering near-perfect concentration estimations with negligible errors for pure (0.35%) and mixtures of 2–4 VOCs (2.0 to 3.5%). The proposed framework, customizable for detecting diverse VOCs and toxic gases, enables scalable indoor and outdoor air-quality monitoring. Its seamless dual functionality ensures uninterrupted wireless communication services during gas sensing, establishing a new paradigm in advanced sensor technology with microwave MIMO E-Nose systems.

## Graphical abstract

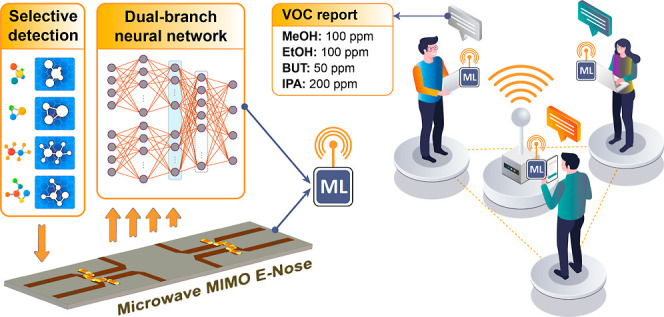

Air pollution remains a leading health threat, responsible for ∼9 million deaths annually,^1^ if no action is taken, its impact is projected to double by 2050.^2,3^ Indoor air, which we inhale ∼90% of the time,^4^ can be up to 10 times more polluted than outdoor air,^5^ causing an estimated 3.8 million premature deaths annually.^6^ Nevertheless, ∼99% of people worldwide continue to breathe air that exceeds safe limits, increasing health risks.^7^ This demands urgent large-scale air quality (AQ) monitoring to identify polluted areas and develop prevention strategies. In reality, over half of low-income countries lack sufficient monitoring stations to report data to the World Health Organization.^8^

To address this, researchers are progressively relying on Internet-of-Things (IoT)-based Wireless Sensor Networks (WSNs) for reliable, cost-effective, and high-resolution real-time AQ monitoring.^9,10^ However, achieving a comprehensive assessment requires several sensors per node, which increases costs and reduces practicality. This creates the need for low-cost, reliable multigas sensors that are scalable and easily integrated into WSNs. Electronic noses (E-Noses) are a popular choice for detecting multiple gases.^11–14^ Recently, E-Nose-based WSNs showed promise for large-scale AQ monitoring.^15^ However, challenges in accurate concentration estimation due to cross-sensitivities and selectivity persist.^16^ Optimizing E-Nose transducers, sensing materials, and algorithms for better accuracy and pattern recognition is crucial. While various transducers have been widely studied for E-Nose applications,^13,17–21^ most fail to meet key WSN integration requirements, such as low power consumption, remote operability, and room-temperature (RT) functionality.

Antenna-based microwave sensors have recently emerged as an ideal solution to meet these requirements. Antenna sensors combine sensing and communication in a compact, cost-effective solution.^22–24^ These systems eliminate the need for separate sensor hardware and simplify integration with radio front-end systems, thereby reducing both the size and complexity of sensor nodes. This makes antenna sensors ideal for real-time wireless monitoring systems, particularly in sensor networks and base stations, where they can replace traditional sensors and antennas. Furthermore, microwave frequency operation enhances sensor performance by speeding up the absorption and desorption of target analytes.

For instance, antenna-based microwave sensors have been used for the selective detection of volatile organic compounds (VOCs) gases.^25,26^ An Ag-coated patch antenna was developed for oxygen detection.^27^ Tin oxide/bionic carbon composites sensitive to ammonia were synthesized in another work.^28^ Additionally, a microwave sensor was fabricated with sensitivity to acetone gas.^29^ To enhance selective detection of gases, antenna sensors incorporating molecularly imprinted polymer (MIP) technology demonstrate significant potential.^30,31^ Multiple antenna elements can be functionalized to achieve chemical selectivity for different gases,^32^ while optimizing sensing materials, structural design, and antenna configuration can enhance both communication and gas sensing performance.^33^ Although MIP-based chemiresistive E-noses have shown promise in detecting gas mixtures,^34^ their seamless integration with existing WSNs still requires additional hardware support. To address this limitation, this research leverages advanced microwave techniques and innovative sensing materials with high selectivity to enable reliable multigas concentration estimation—a critical capability often lacking in traditional antenna sensors. By bridging this gap, the proposed approach offers a more integrated and efficient solution for advanced sensing systems.

This research introduces, for the first time, a dual-functional antenna-based microwave E-nose capable of simultaneous wireless communication and selective detection of VOC mixtures with concentration estimation under ambient conditions. The system effectively addresses cross-reactivity for high sensing performance while employing decoupling circuits to maintain uninterrupted communication. Specifically, we developed MIPs-based sensing materials embedded with multiwalled carbon nanotubes (MWCNTs) to enhance sensitivity and selectivity, thereby reducing cross-reactivity. These optimized sensing structures, functionalized with MIP/MWCNT-based materials specific to target VOCs, were individually tested and integrated into a 4-element multiple-input multiple-output (MIMO) antenna system. A wideband decoupling and matching network (DMN) technique was introduced to minimize mutual coupling (MC) among antenna elements. In parallel, a feature-specific, domain-aware, dual-branch machine learning (ML) model was employed to address cross-reactivity and enable accurate VOC concentration predictions. This study also resolves several technical challenges, including electromagnetic (EM) interference in multielement microwave gas sensing, optimal sensor positioning to reduce interference, and cross-sensitivities. While traditional sensors typically rely on a single response metric, such as resistance changes, the proposed microwave approach exploits both frequency and magnitude responses, reducing false identifications and improving accuracy in gas concentration estimation.

Our efforts culminated in the development of a reliable microwave MIMO E-nose, integrating advanced sensory and data-processing components for highly sensitive detection and accurate concentration estimation of VOCs, both individually and in mixtures, for indoor and outdoor air quality monitoring. This enables comprehensive assessments and addresses critical gaps. We presented a systematic four-stage procedure for design, optimization, and fabrication, paving the way for substantial progress in emerging sensor technology.

## Materials and Methods

### Sensing Material Synthesis

High-purity (99.5%) chemicals, including methanol (MeOH), ethanol (EtOH), butanol (BUT), isopropanol (IPA), poly­(vinyl alcohol) (PVA, 80% hydrolyzed), and glutaraldehyde (GA, 50% wt in water) were sourced from Sigma-Aldrich. MWCNTs, NC7000 were obtained from Nanocyl. MIPs were synthesized for specific target analytes with optimal ratios determined through preliminary tests. PVA served as the polymer, VOCs as template molecules, and GA as the cross-linker (see Figure 1a). Following a green synthesis approach, 50 mg of PVA was dissolved in 3 mL of distilled water (DIW) and ultrasonicated for 30 min. Then, 10 mL of the target VOC was added under magnetic stirring at 65 °C for 1 h. Finally, 50 μL of GA was added, and the mixture was stirred for an additional 3 h, yielding four MIPs, each specific to a VOC. To improve conductivity, MWCNTs were added to the PVA-based MIPs. A secondary solution of MWCNTs (1 mg mL^–1^ in DIW) was prepared and combined with the MIP solutions, followed by overnight stirring at RT. After evaporating the VOCs via thermal treatment on a hot plate under magnetic stirring, the resulting paste-like MIP-MWCNT composite exhibited suitable viscosity and homogeneity for deposition. Using a micropipette, a precise volume of 6 μL of this paste was drop-cast directly onto the IDE region of the antenna structure (see Figure 1b). The paste adhered naturally to the IDE area and was left to dry at RT, forming a uniform sensing layer. This straightforward deposition approach ensured reproducible and stable coatings suitable for sensitive VOC detection.

**Figure 1:**
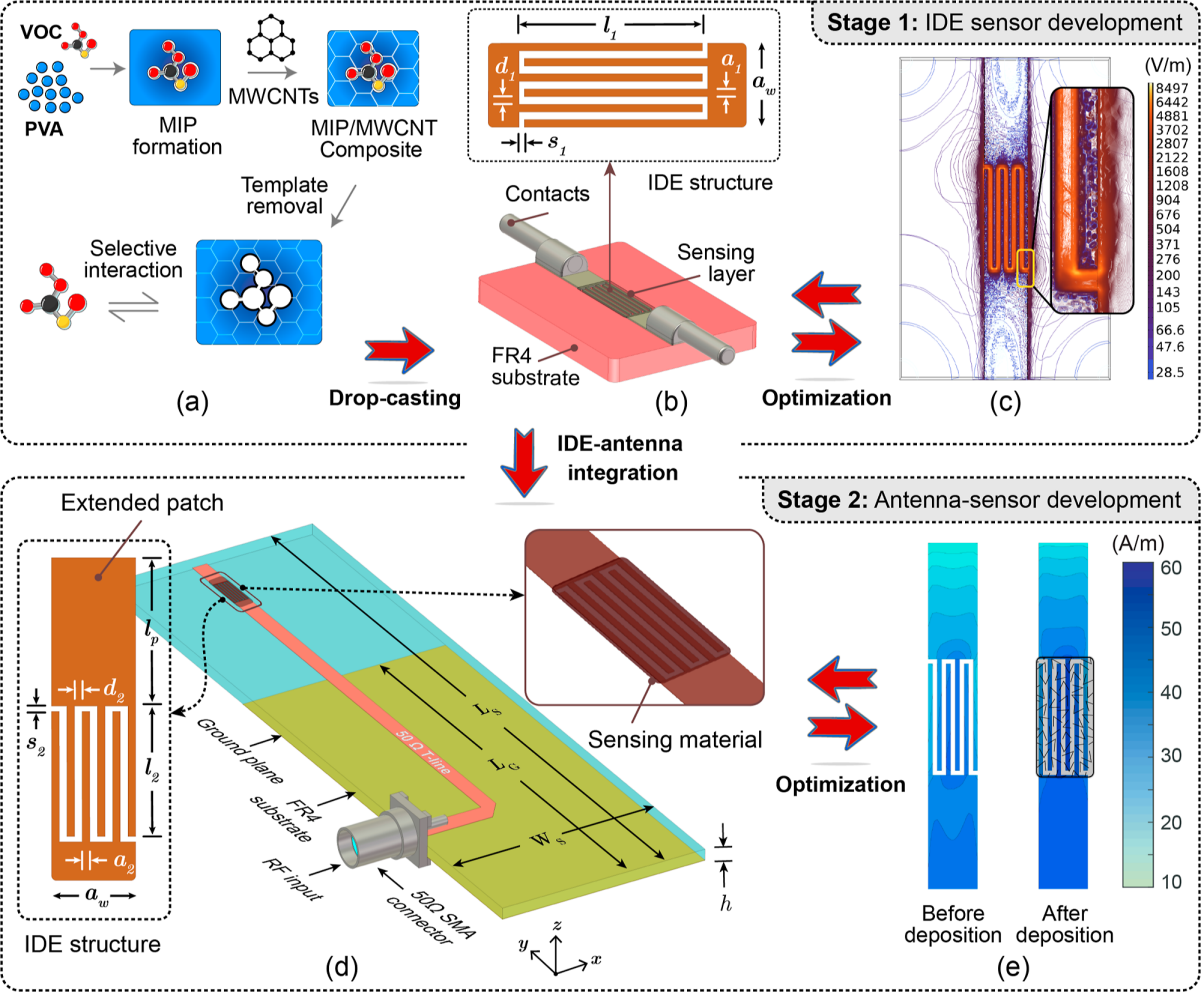
(a) Synthesis process of MIP-based composites, (b) CAD model with geometric parameters of the sensing structure, (c) optimized structure’s electric field distribution, (d) antenna sensor, and (e) current distribution within the antenna’s sensing component.

### Material Characterizations

Scanning electron microscopy (SEM, Mira 3 LMH, Tescan, SE) was performed at an accelerating voltage of 3 kV, a working distance of 15 mm, and a beam intensity of 10 to analyze the microstructure of the composites. Energy-dispersive X-ray spectroscopy (EDS) analysis was conducted on the composites using SEM Mira 3 LMH (Tescan) and Bruker XFlash 6| 10 detector with Esprit 2.1 software. Raman spectra were collected using a Thermo Scientific DXR Raman microscope equipped with a 532 nm line laser. The laser spot size was focused with a 50× objective. The scattered light was analyzed by a spectrograph with holographic gratings of 900 lines mm^–1^ and a pinhole width of 50 μm. The acquisition time was 10 s with 10 repetitions. At a power of 0.3 mW, the spectrum in the black part differed from that in the white part, showing peaks at 354 cm^–1^ and 307 cm^–1^, corresponding to sample crystallization. After increasing the laser power to 1.5 mW, oxidation peaks appeared at 992 cm^–1^ and 818 cm^–1^. Surface chemistry of the composites was assessed using X-ray photoelectron spectroscopy (XPS) on an Omicron Nanotechnology XPS system with monochromatic Al Kα radiation (1486.7 eV). The CasaXPS software was used for spectral deconvolution. X-ray diffraction (XRD) was employed to assess the solid-state properties of the composites using a θ–θ powder diffractometer (X’Pert3 Powder) in Bragg–Brentano geometry with Cu Kα radiation (1.5418 °A, *U* = 40 kV, *I* = 30 mA). Data were collected over an angular range of 5° to 50° (2θ) with a step size of 0.039° (2θ). HighScorePlus 4.0 software was used for diffractogram analysis.

### Microwave MIMO E-Nose Development

This section outlines a four-stage procedure for developing the proposed E-Nose, beginning with Stage 1, which focuses on developing high-sensitivity IDE-based sensing components for optimal performance (see Figure 1a–c). The design parameters were optimized (detailed in Section S2, Supporting Information) to maximize field strength at the electrode gap center, with the structure width (*a*_w_ = 2.35 mm) and patch length (*l*_p_ = 5.0 mm) fixed based on antenna design requirements. The optimized parameters were *a*_1_ = 0.2583 mm, *d*_1_ = 0.16 mm, *s*_1_ = 0.18 mm, and *l*_1_ = 4.82 mm, resulting in a metallization ratio η = 0.62 (defined as the ratio of electrode width to the total pitch). While different parameter combinations can achieve similar field distribution and sensitivity, η significantly impacts electric field strength, gas penetration depth, and measurement sensitivity, directly affecting sensing performance.^35,36^ A 6-electrode copper structure (0.035 mm thick) was fabricated on an FR-4 PCB substrate (1.5 × 1.0 cm) using an LPKF ProtoMat S63 milling machine.

In Stage 2, a single-port antenna-sensor was developed by integrating sensing structures with monopole antenna for gas sensing at 2.45 GHz.^37^ Figure 1d illustrates the 3D design created in Ansys HFSS on an FR-4 substrate (ε_r_ = 4.7, tan δ = 0.02, *h* = 1.55 mm) with copper thickness *t* = 0.035 mm, and dimensions *L*_S_ = 93.29 mm and *W*_S_ = 29.34 mm. The main patch antenna (*a*_w_ = 2.35 mm) was connected to a 50 Ω transmission line fed by a 50 Ω SMA connector on a ground plane (*L*_G_ = 61.63 mm). The IDE structure was coupled to the far end of the patch, enabling gas sensing functionality. The effect of this integration can be analyzed by comparing the antenna’s current distribution with and without the sensing material, as shown in Figure 1e. Minimal current flow was observed through the interdigitated design due to its capacitive nature, with most of the current concentrated in the antenna’s main structure. Coating the electrodes with conductive sensing materials creates additional pathways, extending the current distribution into the extended patch and increasing the antenna’s effective length, which shifts its resonance frequency (see section Coupled E-Nose Response to Single VOCs). To ensure the antenna operates within its bandwidth during sensing, the structure was reoptimized with parameters: *a*_2_ = 0.2583 mm, *d*_2_ = 0.16 mm, *s*_2_ = 0.20 mm, and *l*_2_ = 4.80 mm.

In Stage 3, four single-port antenna sensors were configured into a MIMO system (Figure 2a), each functionalized with a specific sensing material for selective detection of MeOH, EtOH, BUT, and IPA, labeled as Ⓜ, Ⓔ, Ⓑ, and Ⓘ, respectively. The positioning was optimized based on the VOC cross-reactivity profile and element coupling, ensuring spatial isolation while minimizing chemical and EM interferences (see section). The adjacent sensor pairs are symmetrically positioned back-to-back along the *x*-axis, separated by *d* = 0.065λ_0_ mm, ensuring compact design and balanced coupling, where λ_0_ is wavelength in free space. The antennas were initially designed to resonate at 2.45 GHz in isolation. However, in the MIMO configuration, close proximity, shared ground plane, and substrate induced EM field interactions and additional current paths, changing the input impedance and resonance behavior. The design parameters were then fine-tuned to restore resonance at 2.45 GHz.

**Figure 2:**
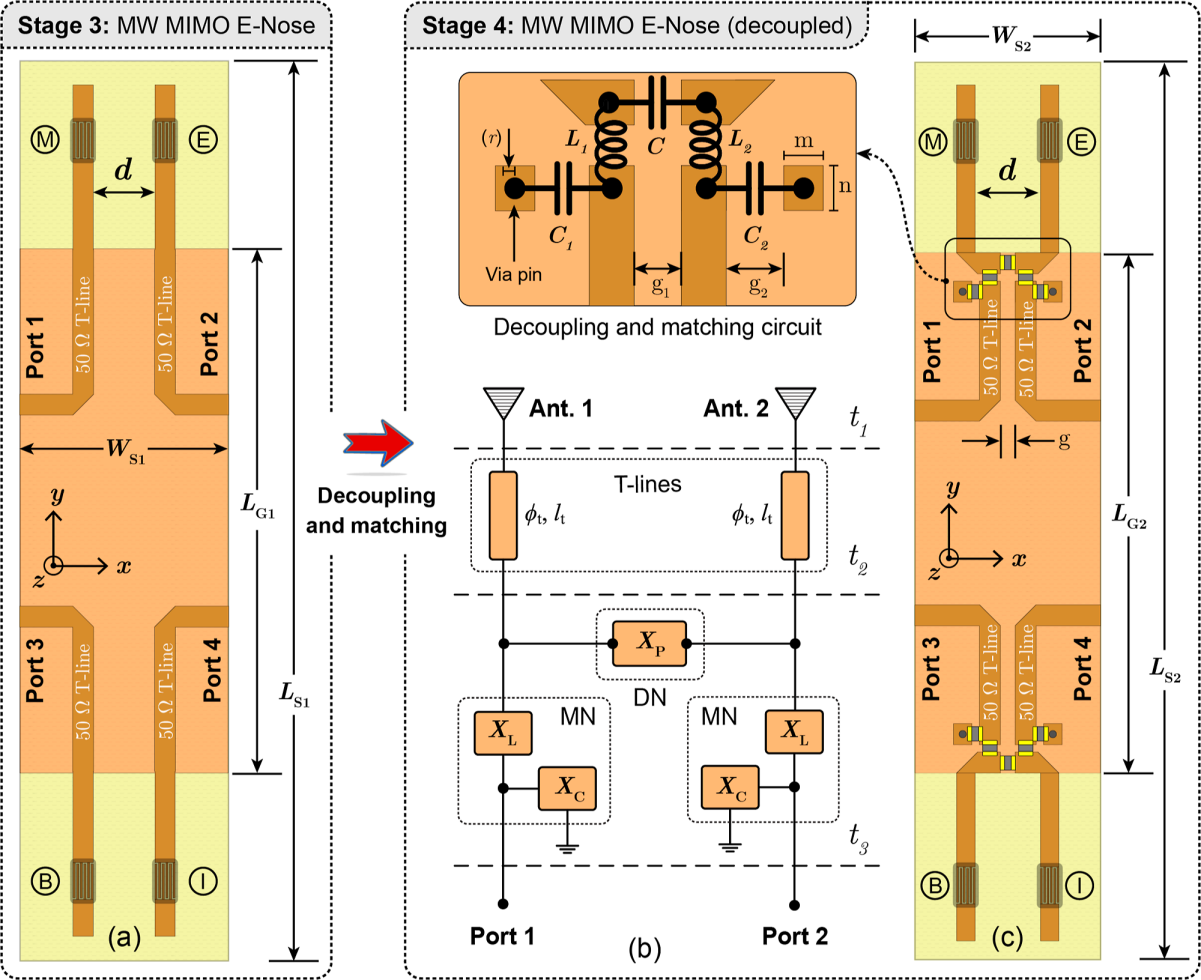
Geometry of the (a) coupled four-port microwave MIMO E-Nose, (b) schematic of the DMN circuit, and (c) decoupled MIMO E-nose.

In the MIMO structure, the modified design parameters were *L*_S1_ = 119.37 mm, *L*_G1_ = 70.68 mm, and *W*_S1_ = 28.29 mm. Mitigating MC is essential for maintaining signal quality, enabling optimal antenna performance, high-speed data transmission, and reliable connectivity in advanced 5G/6G systems. For gas sensing application, MC can interfere with measurements by causing unwanted shifts in the resonance frequencies of adjacent antenna elements, resulting in false detections and inaccurate readings. This complicates the extraction of cross-reactivity information from sensor responses, which is key to accurately distinguish cross-reactive compounds and estimating their concentrations in mixtures. To address this and enable dual functionality using a microwave MIMO E-nose, a low-complexity wideband decoupling technique is employed. In Stage 4, a DMN circuit was designed and integrated into the E-Nose system. Figure Figure 2b details the step-by-step implementation of the decoupling technique (see Section S6 of the Supporting Information).

### VOC Sensing Setup

For gas sensing with the microwave MIMO E-Noses, S-parameters were measured using a four-port Vector Network Analyzer (VNA, Rohde & Schwarz ZVA 67) (see Figure 3). The E-Nose was placed in a 1.0 L gas chamber and connected to the VNA using four 50 Ω SMA cables. The VNA was configured to sweep from 1 to 4 GHz at 100 kHz intervals with a power level of −10 dBm. The VNA collected 30 000 samples during each sweep, with *T*_s_ = 5.0 s. The samples were subsequently processed in MATLAB for signal analysis. Resonance frequencies *f* of the antennas were defined by points indicating the minimum magnitude of the *S* parameter. Precise amounts of liquid VOCs were then evaporated into the chamber using a precision micropipette, with quantities determined based on the ideal gas law. Upon VOC exposure, dynamic changes were observed, and both response time τ_res_ and recovery time τ_rec_ were recorded.

**Figure 3:**
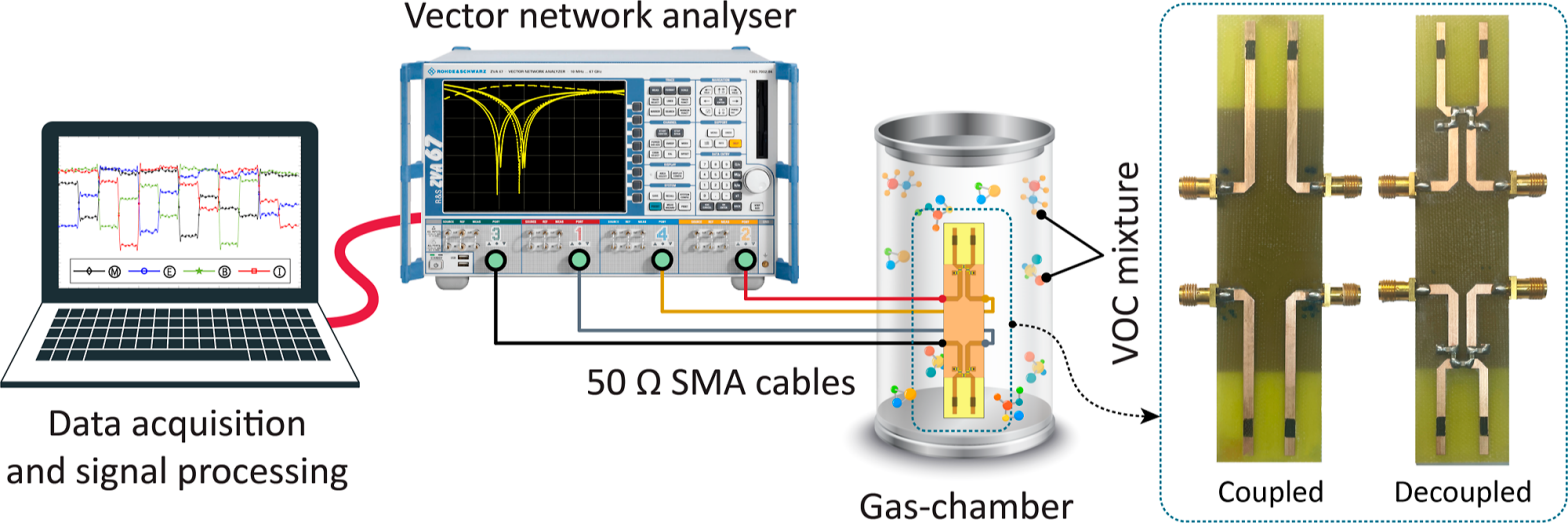
Experimental setup for VOC sensing with microwave MIMO E-nose.

Within each experiment, the concentrations of VOCs in the air chamber varied, except the zero concentrations of all VOCs which were repeated several times due to the experimental setup. The resonance frequencies *f*_*i*_(*k*) and S-parameters magnitudes *S*_*ij*_(*k*) were measured for at least 10 min. Here, *i* and *j* represent the antenna ports, *i*, *j* ∈ {1, 2, 3, 4}, and *k* denotes the sample index. Further, τ_res_, τ_rec_ and concentrations of VOCs were recorded for each experiment. Excluding the all-zero concentrations, measurements were performed for 96 mixtures with varying target VOC concentrations over a span of 30 days. Measurements were conducted under ambient conditions at RT (∼22 °C) and pressure ∼982 mbar with relative humidity between 63 to 66%. For each VOC mixture with at least one nonzero VOC concentration, the frequency and magnitude responses were computed as $\Delta f_{i}\left(k\right)=f_{i}\left(k\right)-{\mathrm{f̃}}_{i}$ and $\Delta S_{ii}\left(k\right)=S_{ii}\left(k\right)-{\mathrm{S̃}}_{ii}$ . Respectively. Here, ${\mathrm{f̃}}_{i}$ and ${\mathrm{S̃}}_{ii}$ denote the initial frequency and initial S-parameter magnitude for the *i*th port, calculated as the mean from the samples of the resonance frequencies *f*_*i*_(*k*) and *S*-parameters magnitudes *S*_ii_(*k*), respectively, recorded for the all-zero VOC concentrations.

### Data Set Preparation

The neural network presented in this work is designed to predict VOC concentrations based on the steady-state responses of the sensors. To achieve this, only steady-state responses were included in the data set **D**, which was prepared for the training and evaluation of the network. The data set **D** consists of 10583 observations, with 90 to 130 observations per experiment. Each observation, indexed as *i*, is represented as a 3-tuples (**F**_*i*_, **S**_*i*_, **C**_*i*_), where $F_{i}={\left[\Delta f_{1}^{j}\left(k\right),...,\Delta f_{4}^{j}\left(k\right)\right]}^{T}$ , $S_{i}={\left[\Delta S_{11}^{j}\left(k\right),...,\Delta S_{44}^{j}\left(k\right)\right]}^{T}$ , $C_{i}={\left[C_{MeOH}^{j},C_{EtOH}^{j},C_{\text{BUT}}^{j},C_{IPA}^{j}\right]}^{T}$ . Here, *j* denotes the experiment number, and *k* represents the sample index, and *i* is the sum of observations in the previous experiments (experiments 1, ..., *j* – 1) plus *k*. The data set **D** was randomly split into training **T** and evaluation **E** data sets with a ratio of 80% (8467 observations) and 20% (2116 observations), respectively.

### Dual-Branch Neural Network for E-Nose

We developed a novel dual-branch neural network to estimate VOC concentrations based on the frequency and magnitude. The network has a depth of four, with fully connected layers and ReLU activation functions incorporated at each level. At the first two levels, the network processes the frequency responses Δ*f*_1_, ..., Δ*f*_4_ and magnitude responses Δ*S*_11_, ..., Δ*S*_44_ in two separate branches, *f* and *m*, respectively (Figure 4). The outputs of these branches, **x**_f_^(2)^ and **x**_m_^(2)^, are concatenated and subsequently processed by the third and fourth dense layers. The number of neurons *N* in the hidden layers is defined as *N*_f_^(1)^ = 64, *N*_f_^(2)^ = 32, *N*_m_^(1)^ = 32, *N*_m_^(2)^ = 16, *N* ^(3)^ = 64, where the superscripts indicate the layer index. A dropout layer with a dropout probability of 0.2 is applied exclusively at the output of the third layer.

**Figure 4:**
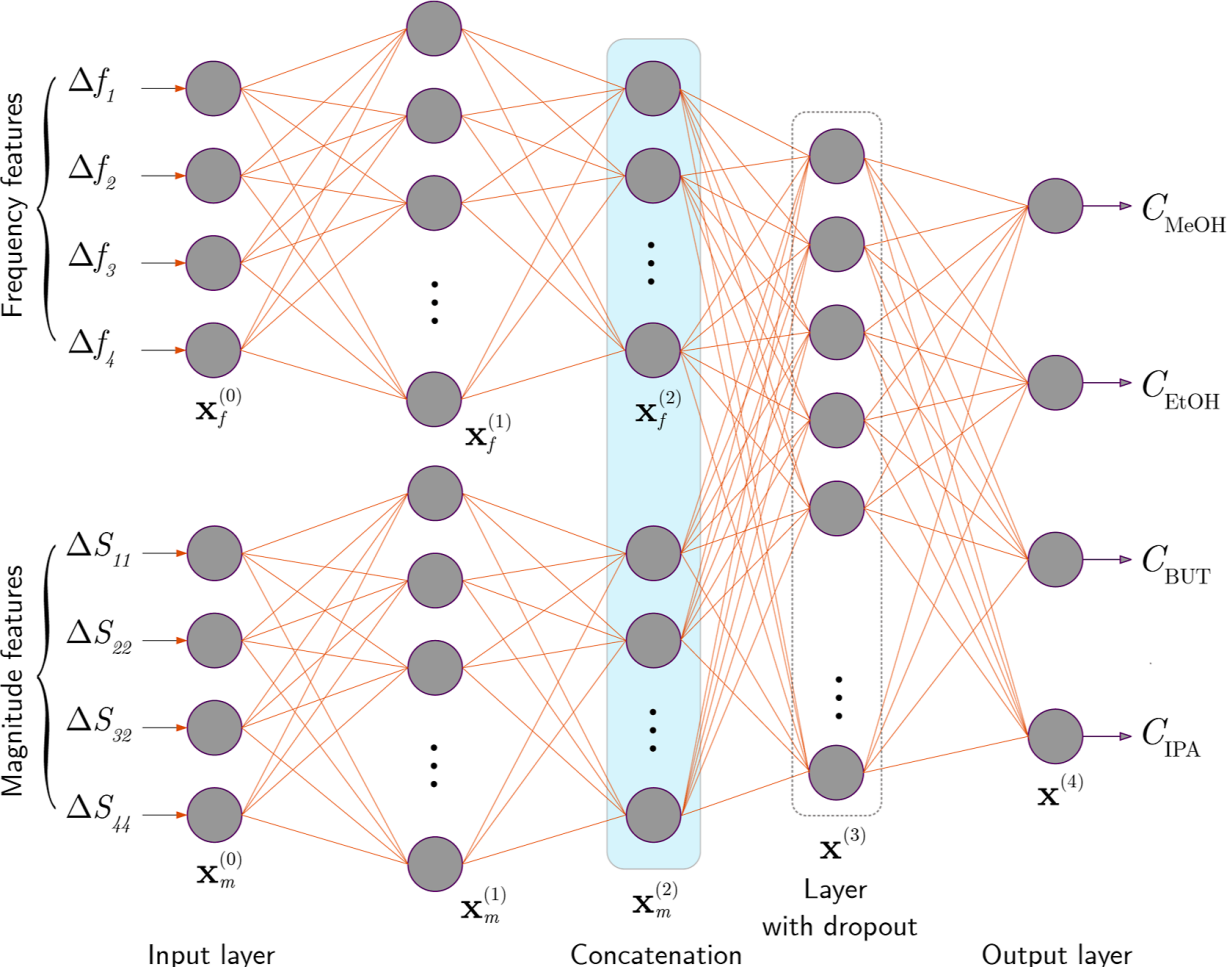
A dual-branch NN architecture for VOC concentration estimation, designed to separately process frequency and magnitude features, enabling feature prioritization and utilizing insights from transducer responses. Here. **x** ^(*i*)^ represents the neurons in the *i*th layer, with subscripts *f* and *m* denoting the frequency and magnitude branches, respectively.

Since the magnitude responses are prone to noisy or missing data, whereas the frequency responses are not, different treatments of the weights in the magnitude and frequency branches should be applied during the training phase. To address this, L1 regularization is employed in the magnitude branch to encourage sparsity and robustness, while L2 regularization is applied in the frequency branch to reduce the influence of less significant features without completely eliminating them. As a result, the loss function used during training is defined as

1 $$\mathrm{L̃}=L+\alpha_{1}\left(∥W_{m}^{\left(1\right)}{∥}_{1}+∥W_{m}^{\left(2\right)}{∥}_{1}\right)+\alpha_{2}\left(∥W_{f}^{\left(1\right)}{∥}_{2}^{2}+∥W_{f}^{\left(2\right)}{∥}_{2}^{2}\right)$$

where *L* is a nonregularized loss function **W**_m_^(i)^ and **W**_f_^(i)^ represent the weights in the *i*th layer of the magnitude and frequency branches, respectively. The regularization strengths α_1_ and α_2_ were set to 0.02 and 0.01, respectively.

Since the domain of the network outputs is continuous, the mean squared error (MSE)

2 $$MSE=\frac{1}{\left|\Gamma \right|N}\sum_{\forall j\in \Gamma }\;\sum_{i=1}^{N}{\left(C_{ji}-{Ĉ}_{ji}\right)}^{2}$$

and mean absolute error (MAE)

3 $$MAE=\frac{1}{\left|\Gamma \right|N}\sum_{\forall j\in \Gamma }\;\sum_{i=1}^{N}\left|C_{ji}-{Ĉ}_{ji}\right|$$

are the performance measures of the choice. Here, *N* is the number of samples used for evaluation, *C*_*ji*_ and ${Ĉ}_{ji}$ represent the true and predicted concentrations of the *j*th VOC in the *i*th sample, and Γ = {MeOH, EtOH, BUT, IPA}.

The proposed network was trained on the training data set **T**, minimizing the regularized loss function (1) with MSE (2) as *L*. Prior the training, the model parameters were initialized with random values sampled from a uniform distribution. The parameters were updated using the Adam optimizer with a learning rate of 0.01 and exponential decay rates of 0.9 and 0.999 for the first and second moment estimates, respectively. Training was performed with a batch size of 32 samples, and the data was shuffled at the beginning of each epoch. The hyperparameter configuration was determined through grid search (see Section S3 of the Supporting Information).

The evaluation of the trained model was performed on the evaluation data set **E** using MSE (2), MAE (3) and the *R* ^2^ coefficient. Due to the varying evaporation times of liquid VOCs, the number of steady-state samples per experiment ranged from 90, to 130, resulting in an imbalanced data set. To preserve the proportional distribution of observations across experiments, stratified splitting was applied during both the training and evaluation phases.

To account for different grouping criteria, the percentage average prediction error for the *j*th VOC was calculated as

$$\varepsilon_{j}^{\left(p\right)}=\frac{1}{N_{p}}\sum_{i=1}^{N_{p}}\left|\left(C_{ji}^{\left(p\right)}-{Ĉ}_{ji}^{\left(p\right)}\right)/C_{ji}^{\left(p\right)}\right|\times 100\%$$

where *p* represents the grouping criterion, *N*_p_ is the total number of samples in the *p*th group, *C*_*ji*_^(p)^ is the true value, and ${Ĉ}_{ji}^{\left(p\right)}$ is the predicted value of the *i*-th sample in the group *p*. The groups were based on concentration levels (*p* ∈ {1000, 2000, ..., 10000}) and mixture complexity levels (*p* ∈ {1, 2, 3, 4}).

## Results and Discussion

### Material Characterizations

Since the polymer and MWCNTs play distinct yet equally important roles in forming the composite film, achieving a homogeneous distribution of both materials is essential. Therefore, examining the surface morphology of the synthesized composites is crucial for assessing the dispersion of nanotubes within the polymer matrix. SEM images of the synthesized MIP/MWCNTs composites are presented in Figure 5. It can clearly be seen that all composites exhibit a uniform and smooth surface morphology, as shown in Figure 5a–d. Moreover, cross-sectional images confirm the uniform formation of the composites, with the MWCNTs well-incorporated into the MIP matrix (see Figure 5e–h).^38,39^ Elemental ratios of the fabricated samples were investigated using EDS analysis, as shown in Figure 6a. C and O peaks were observed in the EDS spectra for all samples. The differences observed in the C/O ratios (Figure 6b) of the synthesized samples may be attributed to the solvents used during the synthesis of MIPs.

**Figure 5:**
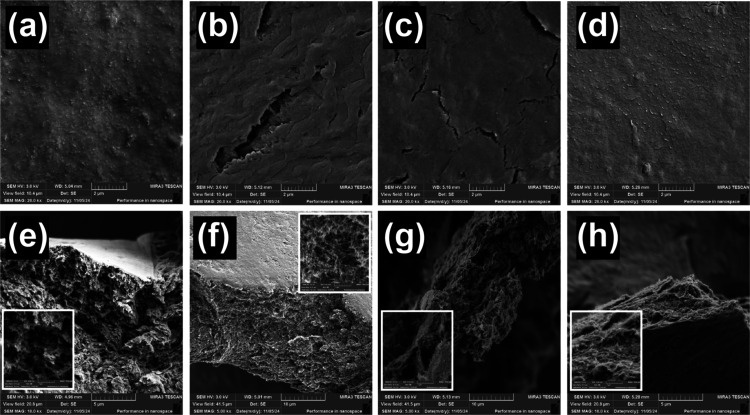
SEM images of MIP composites synthesized using different solvents: (a) MeOH, (b) EtOH, (c) BUT, and (d) IPA. Panels (e–h) show corresponding cross-sectional views obtained by mechanically scratching the composites, with higher magnification insets.

**Figure 6:**
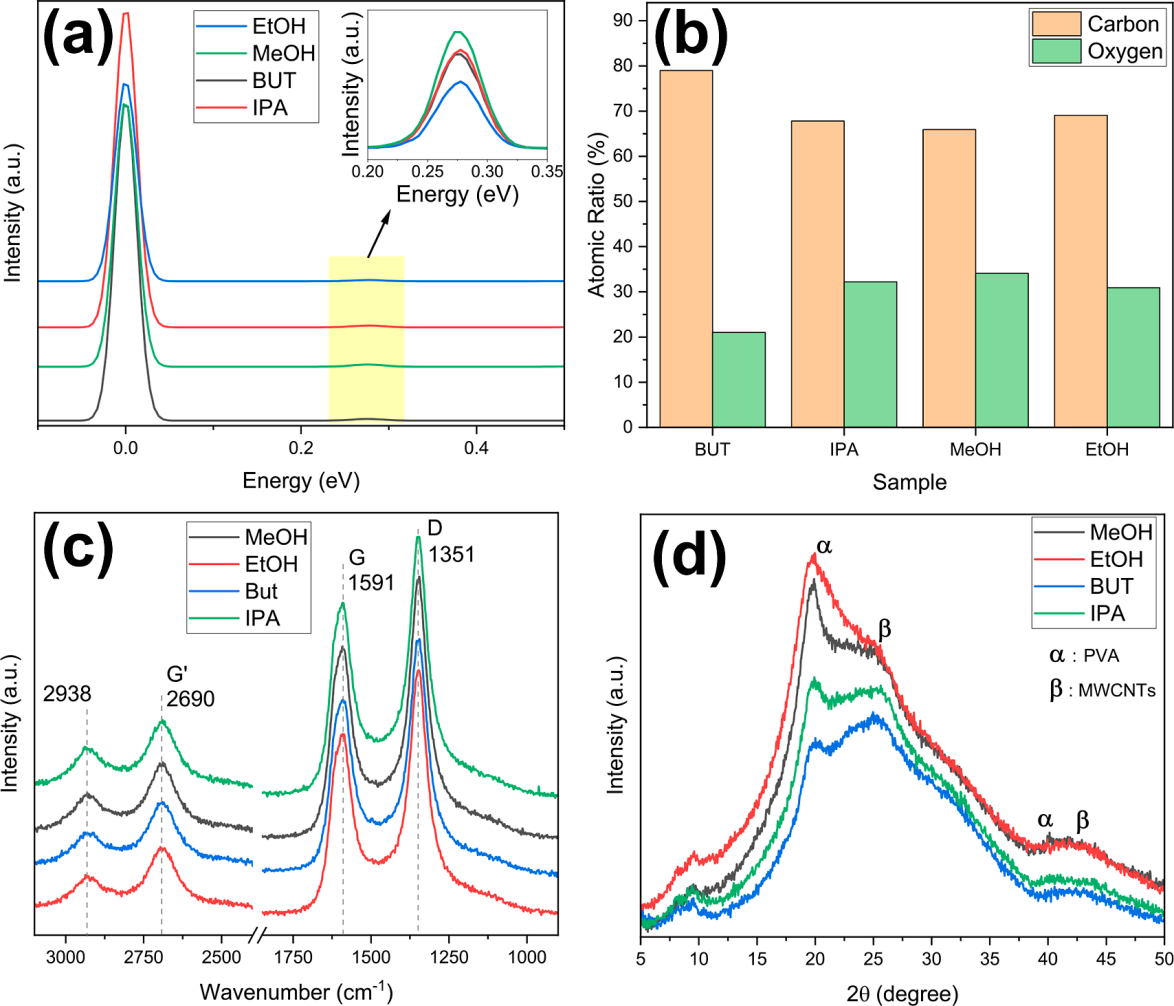
(a) EDS spectra, (b) EDS O/C ratios, (c) RAMAN spectra, and (d) XRD spectra of MIP/MWCNTs composites.

Raman spectroscopy was used for the structural characterization of MIP-based composites. Figure 6c shows the Raman spectra of four MIP/MWCNT composites. Three characteristic peaks related to the MWCNTs were observed at 1351 cm^–1^, 1591 cm^–1^, and 2690 cm^–1^, corresponding to the D, G, and G′ bands, respectively. The D band is associated with structural defects, the G band corresponds to ordered carbon domains, and the G′ band is related to disordered graphite. Additionally, the peak observed at 2938 cm^–1^ is attributed to the –CH_2_ stretching vibration in the PVA molecule. No peak shifts were observed in samples imprinted with different VOCs.^40–42^ The crystal structure of the synthesized composites was investigated by XRD (Figure 6d). The peaks at 25.54° and 42.30° correspond to the (002) and (101) planes of MWCNTs, respectively. The peaks at 19.85° and 50.52° are related to PVA.^40,43^ Despite using different solvents for each MIP, the synthesized composites were structurally identical, indicating that similar structures were obtained by imprinting against different VOCs using the same method.

The chemical composition of the synthesized composites was identified using the XPS spectra of the O 1s and C 1s regions. As shown in Figure 7a, the C 1s spectra consist of three distinct peaks: the peak at ∼284.6 eV, associated with hydrocarbon species; the peak at ∼288.8 eV, related to carboxylate carbon; and the peak at ∼286 eV, attributed to C–OH and O–C–O bonds.^44,45^ The O 1s spectra were deconvoluted into two components: the peak at ∼531.5 eV, corresponding to C=O bonds, and the peak at ∼532.8 eV, associated with hydroxyl groups as seen in Figure 7b.^46,47^ The compositional ratios deduced from the peak areas are presented in Table 1. The similarity in component ratios across all four samples indicates that these materials share similar structures.

**Figure 7:**
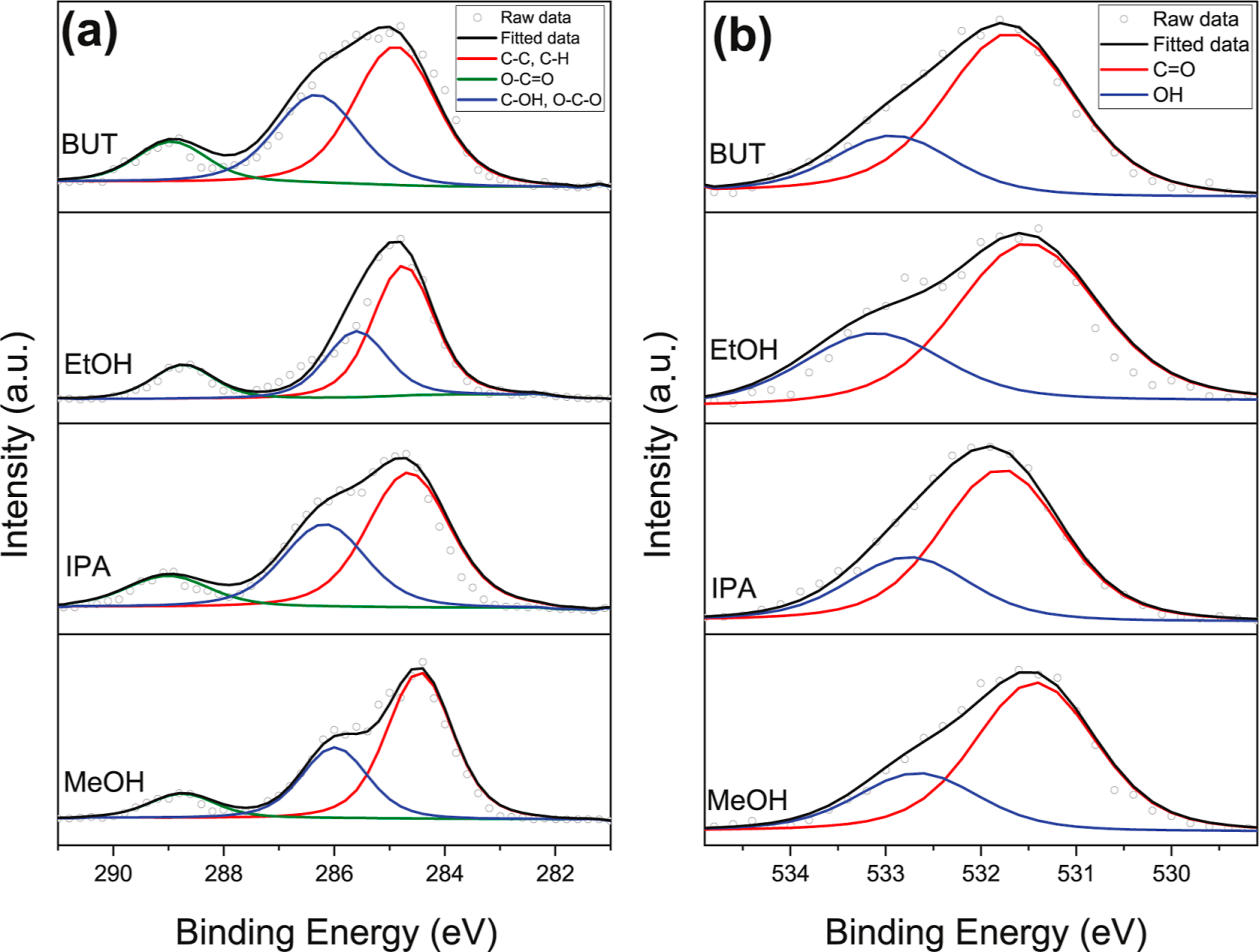
XPS (a) C 1s spectra, and (b) O 1s spectra.

**Table 1:** XPS Ratios of Chemical Bonds and Elements

	C 1s (%)			O 1s (%)	
sample	C–C, C–H	C–OH, O–C–O	O–C=O	C=O	OH
MeOH	33.6825	16.2358	5.51257	32.1898	12.3792
EtOH	34.0152	17.2806	8.80791	27.5033	12.3929
BUT	27.8717	17.6636	6.85233	36.3244	11.288
IPA	30.7405	18.8112	6.94741	30.7257	12.7752

To further investigate the swelling mechanism between the target analyte and the MIP matrix, FT-IR spectroscopy was performed on the MeOH-selective MIP film before and after exposure to increasing concentrations of MeOH vapor. Figure S1 in the Supporting Information presents the FT-IR spectra of the MIP film under four conditions: prior to MeOH exposure, after exposure to 5000 ppm and 10,000 ppm MeOH, and after the recovery process. Characteristic absorption peaks and their associated chemical groups are labeled in the Figure S1.^33^

A clear and progressive increase in peak intensities was observed with increasing MeOH concentration, indicating methanol adsorption. These increases in the FT-IR spectra can be attributed to the swelling of the polymer matrix, consistent with similar reports in the literature.^48,49^ In particular, the observed increases in peaks associated with −OH stretching at 3331 cm^–1^, C–H stretching at 2945 cm^–1^ and 2836 cm^–1^, and C–O stretching at 1101 cm^–1^ and 1020 cm^–1^ further support the interaction between MeOH and the functional groups in the MIP.^50,51^ Notably, the peak intensities return to their baseline values after the recovery process, confirming the reversibility of the adsorption.

This spectral evolution supports the hypothesis of polymer swelling and enhanced dipole–dipole interactions upon VOC exposure, providing indirect yet compelling evidence of the molecular recognition and swelling mechanism of the MIP toward its target VOC.

### Response Analysis: Sensing Components

The sensing elements were tested individually (see Section S3 of the Supporting Information) prior to integrating the IDE antenna for two key reasons: first, to assess their performance in detecting target VOCs, as the E-Nose’s gas detection efficiency depends on them; and second, to identify primary and secondary cross-reactive compounds for each sensing component, optimize antenna sensor positioning, and extract cross-sensitivity information while minimizing EM interference. This understanding enables the strategic positioning of antenna sensors to achieve spatial isolation and reduce coupling effects on gas sensing, thereby improving measurement accuracy (see Section S4 of the Supporting Information).

### E-Nose Antenna Performance: Coupled/Decoupled

Figure S1 (in the Supporting Information) presents the measured S-parameters of the E-Nose with and without the decoupling circuit and before and after sensing material deposition. Initially, the antenna elements resonated at 2.452, 2.470, 2.467 and 2.462 GHz (Figure S1a), slightly deviating from the design frequency of 2.45 GHz, likely due to milling machine tolerances. After sensing material deposition, the resonance shifted to 2.361, 2.381, 2.376 and 2.371 GHz, as the conductive coating increased the antenna’s effective length (see section Microwave MIMO E-Nose Development). The coupled E-Nose achieved a measured 10 dB bandwidth of approximately 630 MHz, which remained unchanged after deposition.

The back-to-back symmetric arrangement and close proximity of the antenna elements result in significant coupling between the ports. The strongest coupling (−3.5 dB) occurs between adjacent antenna pairs (Port 1–2 and Port 3–4), as shown in Figure S1b. This strong mutual coupling severely degrades the antenna’s efficiency, bandwidth, and overall transmission performance.^52^ Furthermore, this can significantly impact gas sensing performance—a challenge not encountered in traditional sensing methods and, therefore, not previously addressed in the literature.

The decoupled E-Nose achieved a slightly narrower bandwidth of ∼511 MHz (Figure S1c). However, the decoupling technique provides a ∼315 MHz 15 dB isolation bandwidth, equivalent to a relative isolation bandwidth of 12.8%. This 10 dB operational bandwidth of 511 MHz with 12.8% isolation bandwidth ensures simultaneous, uninterrupted wideband wireless communication and gas sensing between adjacent antenna pairs.

The radiation patterns of the E-Nose were evaluated at 2.45 GHz in an anechoic chamber before and after coating with the sensing material, showing no significant changes in radiation characteristics, efficiency, or gain (see Figure S2 in the Supporting Information). The E-Nose exhibits an omnidirectional radiation pattern, radiating uniformly in all directions perpendicular to the antenna plane, suitable for 360-degree communication in applications like mobile devices and Wi-Fi routers. A slight tilt in the pattern arises from the finite size of the monopole’s ground plane. While reactive components introduce minor losses, wideband isolation and matching techniques minimize performance degradation. The E-Nose achieves a peak gain of 8.20 to 8.25 Bi and a total radiation efficiency of 80.0 to 81.5%. Measurements were performed with one port active and others terminated with 50 Ω loads to ensure accuracy.

### Coupled E-Nose Response to Single VOCs

To demonstrate the effect of EM interference on cross-reactivity, the coupled E-Nose was tested with VOCs at 1000 to 5000 ppm. Upon exposure to MeOH, Ⓜ showed a linear frequency shift with increasing concentration (∼729 kHz/1000 ppm). However, Ⓔ also exhibited a significant response due to a high transmission coefficient (*S*_12_) of −3.5 dB and cross-reactivity between Ⓜ and Ⓔ (Figure S4 in the Supporting Information). For example, when exposed to MeOH at 5000 ppm, Ⓜ and Ⓔ responded with 3.645 and 1.30 MHz, respectively, indicating a false detection of a VOC mixture containing MeOH (5000 ppm) and EtOH (1993 ppm). Similar issues arose between Ⓑ and Ⓘ due to high *S*_34_ (−3.5 dB). Such false detections can be mitigated by analyzing cross-sensitivity patterns. However, strong coupling between these elements complicates the extraction of meaningful data, requiring decoupling techniques.

### Optimal Sensor Placement in E-Nose

The decoupling circuit effectively isolates E-Nose response pairs Ⓜ–Ⓔ and Ⓑ–Ⓘ, achieving a 12.8% 15 dB isolation bandwidth (see Figure S4 in the Supporting Information), enabling cross-reactivity analysis without EM interference. With this wideband isolation, sensors at Ports 1–2 and 3–4 were functionalized to detect two VOC pairs with primary cross-reactivity: MeOH–EtOH and BUT–IPA (see Figure S1 in Supporting Information). While the opposite antenna pairs (Port 1–3 and Port 2–4) showed moderate coupling (−17.9 dB), the diagonal ports (Port 1–4 and Port 2–3) exhibited the weakest coupling (−22.1 dB) (see Figure S1b). To exploit the mutual admittance between these two pairs, the sensing components with the first secondary cross-sensitivities (Ⓜ–Ⓘ and Ⓔ–Ⓑ) were positioned diagonally, while the components with the next secondary cross-sensitivities (Ⓜ–Ⓑ and Ⓔ–Ⓘ) were positioned on opposite sides.

The goal was to design a layout that enables accurate analysis and extraction of cross-reactivity relationships between VOCs with minimal EM interference. This strategic positioning improves the E-Nose’s selectivity and accuracy, balancing interactions between compounds with varying cross-reactivity. The final resonance frequencies of decoupled Ⓜ, Ⓔ, Ⓑ, and Ⓘ were 2.339, 2.359, 2.364, and 2.389 GHz (see Figure S3 in the Supporting Information) and recorded as baseline frequencies (*f*_0_) prior to VOC exposure.

### Decoupled E-Nose Response to Single VOCs

Gas sensing performance was evaluated by exposing the decoupled E-Nose to pure VOCs at 1000 to 5000 ppm (see Figure 8). Ⓜ, Ⓔ, Ⓑ, and Ⓘ demonstrated rapid detection within τ_res_ = 1.5 to 3.5 min, exhibiting strong selectivity toward their respective target VOCs. The Δ*f* responses confirmed a linear detection proportional to concentrations with sensitivities of 669, 660, 848 and 754 kHz per 1000 ppm, respectively. The mean sensitivity (σ_*i*_) for the *i*-th gas was calculated as follows^37^

4 $$\sigma_{i}=\frac{1}{N}\sum_{k=1}^{N}\left(\Delta f_{i}^{\left(k\right)}/C_{i}^{\left(k\right)}\right)\left(Hz/ppm\right)$$

where *C* ^(*k*)^ denotes *k*th concentration of the VOC.

**Figure 8:**
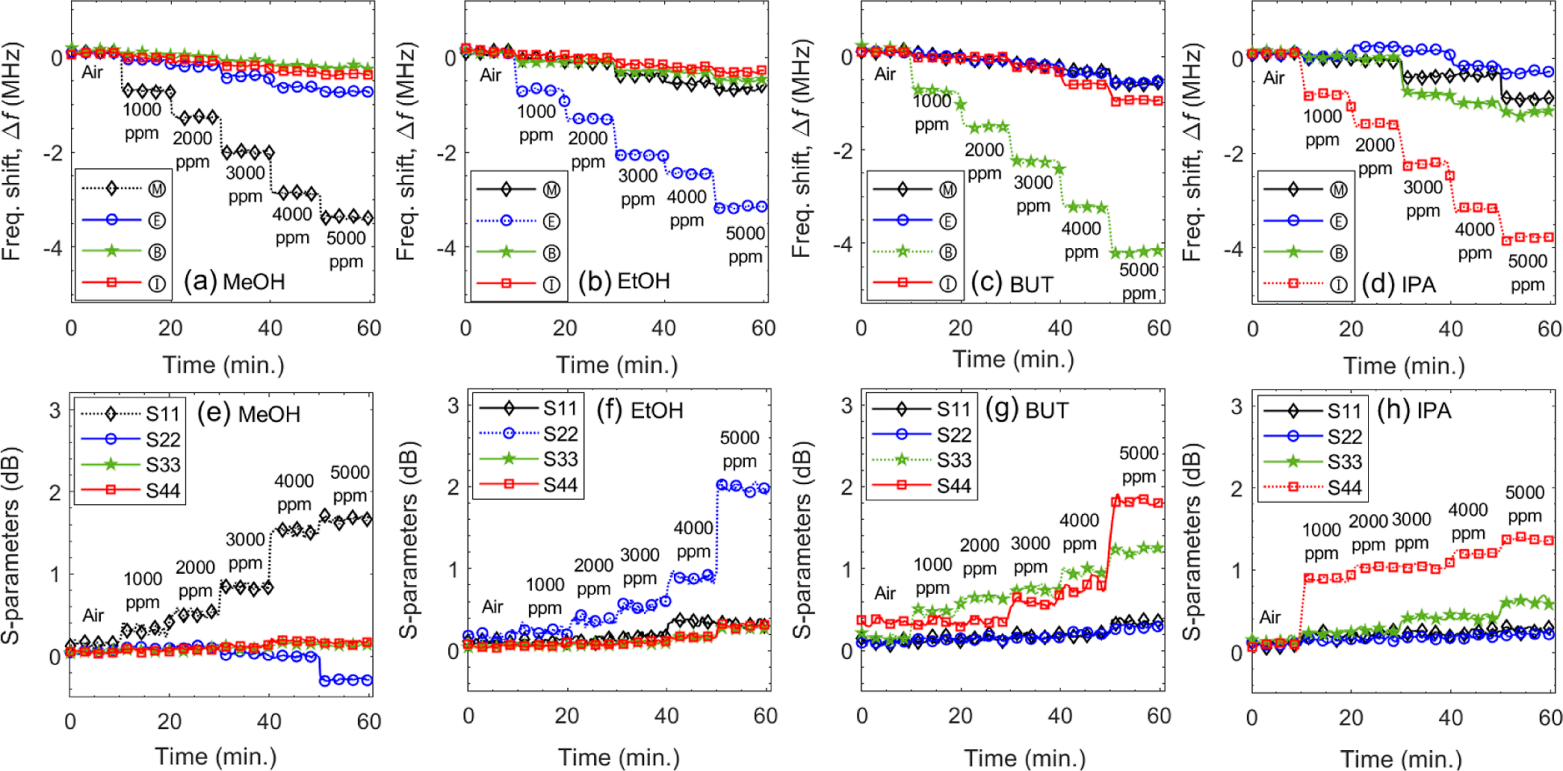
Decoupled E-Nose response (Δ*f*, Δ*S*) during gas exposure for pure (a,e) MeOH, (b, f) EtOH, (c, g) BUT, and (d,h) IPA at 1000 to 5000 ppm, showing varying cross-sensitivity levels and identifying primary and secondary response features for each VOC.

The permissible exposure limit (PEL) varies among VOCs according to their toxicological profiles.^53^ Therefore, the sensor must achieve a detection limit (DL) significantly below each VOC’s PEL to enable early and reliable detection in safety-critical environments. To evaluate this, semiempirical DLs were estimated from the measured concentration ranges using polynomial fitting and calibration curves (see Figures S6 and S7 in the Supporting Information), following the methodology outlined in refs 37,54, and 55 . The calculated σ_*i*_ and DL values are summarized in Table 2, confirming highly sensitive detection and compliance with clean air safety standards.

**Table 2:** E-Nose Response Overview: Cross-Reactive Compounds, Measured Concentration, σ_*i*_, DLs, PELs

sensor	target	cross-reactive VOCs	conc. (ppm)	σ_*i*_ (Hz/ppm)	DL (ppm)	PEL (ppm)
Ⓜ	MeOH	EtOH > IPA > BUT	1000	690	76.3	200
Ⓔ	EtOH	MeOH > BUT > IPA	1000	628	91.746	1000
Ⓑ	BUT	IPA > EtOH > MeOH	1000	914	29.26	200
Ⓘ	IPA	BUT > MeOH > EtOH	1000	784	38.3	400

Unlike CR sensors, which in general only use Δ*R* for gas identification and quantification, the proposed microwave MIMO E-Nose exploits both Δ*f*_*i*_ and Δ*S*_ii_ responses. This dual-metric approach significantly reduces false identifications and enhances the accuracy and reliability of concentration estimations. The (*S*_ii_) responses showed a distinct response for target VOCs, although nonlinearly. Additionally, at least one cross-sensitive response was observed when exposing the sensor to BUT and IPA, while MeOH and EtOH exhibited low/negligible cross-sensitivities. However, with the frequency response, strong cross-reactive responses (at varying levels) were observed for all VOCs. For instance, when exposed to pure MeOH of 5000 ppm, the E-Nose showed an array response of 3.36, 0.739, 0.159 and 0.363 MHz. This indicates a mixture of MeOH (5000 ppm), EtOH (1133 ppm), BUT (188 ppm), and IPA (480 ppm). Similar cross sensitivities were also observed when the E-Nose was exposed to other VOCs. Without addressing this issue, E-Nose can lead to inaccurate readings. Cross-reactive compounds for each sensing component were identified, ranked by interference level (e.g., primary, secondary), and summarized in Table 2.

The Spider chart illustrates the normalized E-Nose responses to target VOCs, showing both selectivity and cross-reactivity (see Figure 9a). Each axis represents a VOC, with radial distances indicating average normalized sensor responses. Greater distances from the center denote stronger responses to specific gases. Distinct shapes and spreads demonstrate the system’s strong selectivity and specificity, while minimal spread toward other VOCs indicates low, yet distinct, cross-reactivity. Overlapping responses suggest that while the E-Nose can selectively detect multiple gases, the responses are not entirely independent, complicating discrimination and estimation in mixtures. This highlights the need for ML techniques to improve accurate VOC distinction.

**Figure 9:**
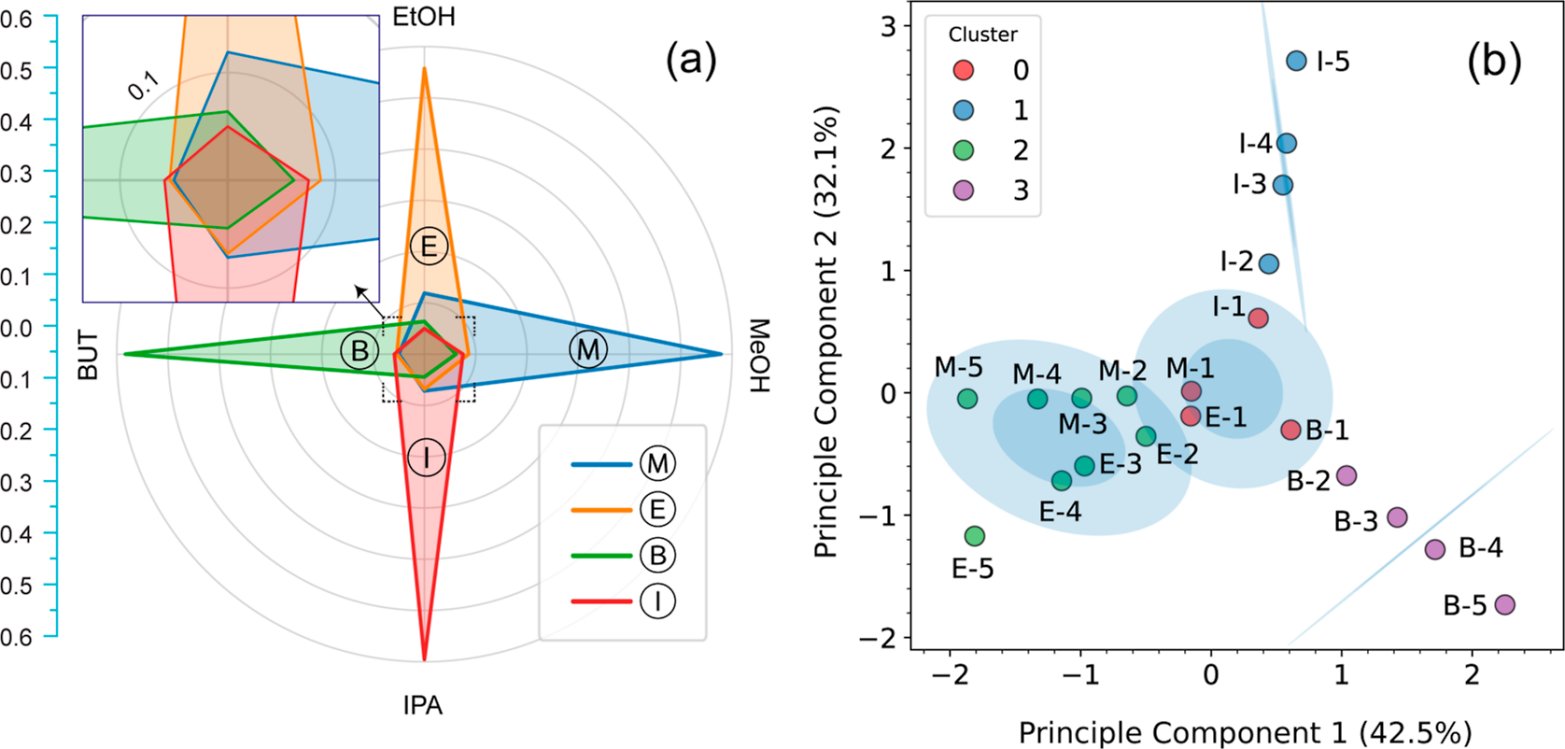
(a) Spider chart comparing the selectivity and cross-reactivity of different sensor responses. (b) PCA-based clustering with K-means and cluster ellipses, showing overlap at lower concentrations and distinct clusters at higher concentrations.

Principal Component Analysis (PCA) was used to assess the variance and clustering of E-Nose responses to various VOCs across all concentration levels. The PCA plot reveals key insights into the E-Nose’s performance, guiding sensor development. Clusters for each VOC are labeled by gas type and concentration (e.g., “M – 1” for MeOH at 1000 ppm). At concentrations above 3000 ppm, clusters are well-separated, indicating strong selectivity and minimal cross-reactivity. Below 3000 ppm, clusters overlap, particularly among similar VOCs like MeOH and EtOH, suggesting higher cross-reactivity and reduced selectivity. This highlights the E-Nose’s high selectivity at higher concentrations but shows the need for advanced machine learning techniques to optimize VOC identification and concentration estimation at lower concentrations. Moreover, the first two principal components explain 74.6% of the total variance (PC1:42.5%, PC2:32.1%), capturing dominant trends driven by both selectivity and cross-reactivity in the sensor responses. The remaining variance reflects nonlinear interactions and cross-sensitivity effects across the sensing array. Thus, while PCA aids in visualizing broad separation trends, accurate VOC identification and quantification require machine learning models that exploit the full multidimensional response space without dimensionality reduction.

### Sensing Mechanism

The analyte recognition ability of MIPs is well-documented in the literature, with thermodynamic principles describing how selective binding and VOC adsorption induce polymer swelling, resulting in volumetric expansion (Figure 10a–d).^43,56–58^ This expansion moves the embedded nanotubes apart (Figure 10c), reducing conductive pathways and increasing the sensor’s resistance.^59,60^ This further changes its effective permittivity,^61^ leading to a change in the reactance.^62–65^ To observe this in our case, SEN1 was exposed to 5000 ppm MeOH. This changed sensor’s resistance from 444 Ω to 532 Ω and the reactance from −78 Ω to −127 Ω (see Figure 10e,f). Therefore, the sensing element functions as a concentration-controlled variable impedance device that selectively responds to its target VOC only.

**Figure 10:**
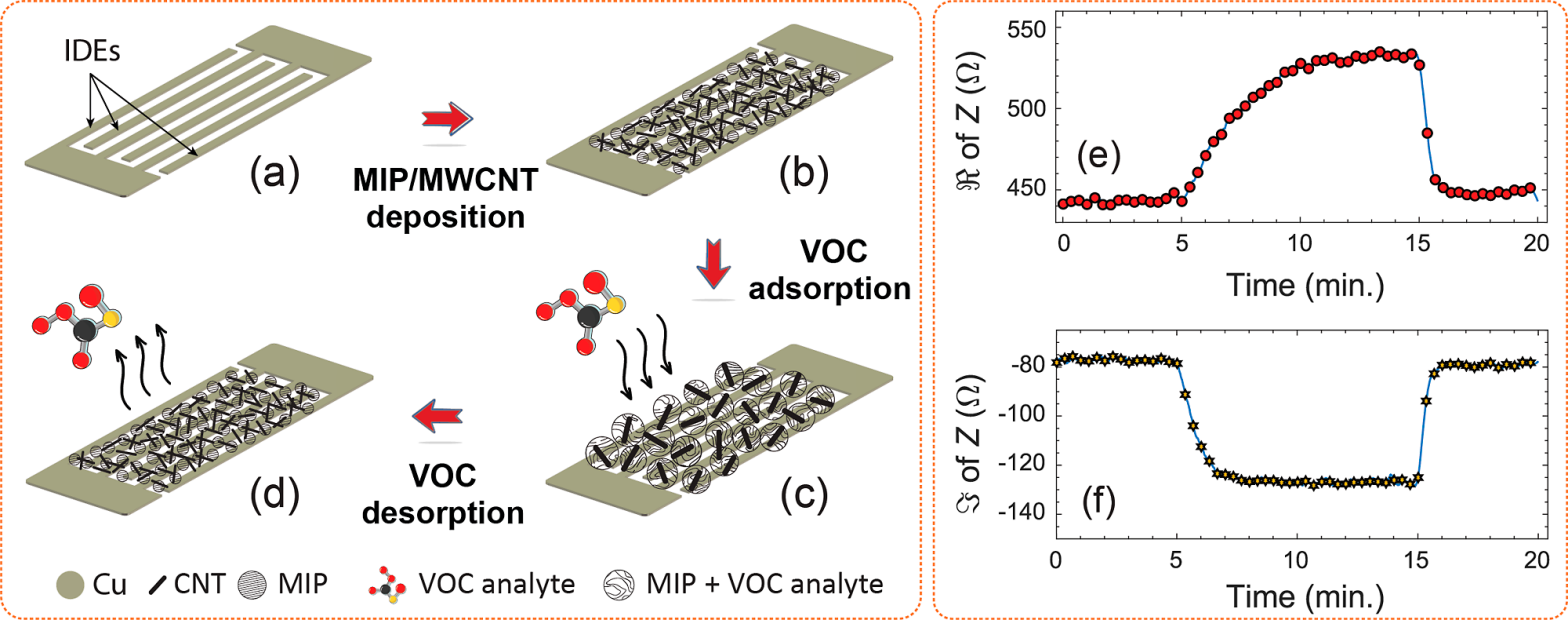
(a,b) MIP/MWCNT-coated IDE structure and its sensing mechanism during (c) gas absorption and (d) desorption, along with changes in (e) real and (f) imaginary impedance.

With IDE-antenna integration, this impedance is coupled with the antenna load impedance (*Z*_L_), changing the S-parameters as *S*_ii_ = (*Z*_L,*i*_ – *Z*_0_)/(*Z*_L,*i*_ + *Z*_0_). This initially shifts the antenna’s resonance (see Figure S1a,c in the Supporting Information), compensating for changes. The antenna’s resonance frequency depends on the balance between inductive and capacitive reactances and is given by $f_{r}=1/\left(2\pi \sqrt{LC}\right)$ , where *L* and *C* represent the inductance and capacitance of the antenna. Integrating a reactance-varying component causes the antenna’s resonance to shift as a function of VOC concentration. This frequency shift can be used to accurately and selectively indicate the presence and concentration of the gas, forming the core principle of this work. Moreover, the extent of the frequency shift upon gas exposure can be optimized for specific wireless applications by optimizing the sensing structure. This enables selective, continuous gas sensing while maintaining uninterrupted communication services simultaneously.

### VOC Mixture Sensing with E-Nose

In mixtures, VOCs coexist without reacting chemically under ambient conditions, preserving their distinct properties and concentrations. An E-Nose should ideally detect each VOC separately, generating responses similar to single-VOC measurements. In practice, however, the E-Nose records stronger responses to mixtures due to both target VOCs and cross-reactive interference, with response complexity increasing as the number of VOCs rises, as shown in Figure 11a–c.

**Figure 11:**
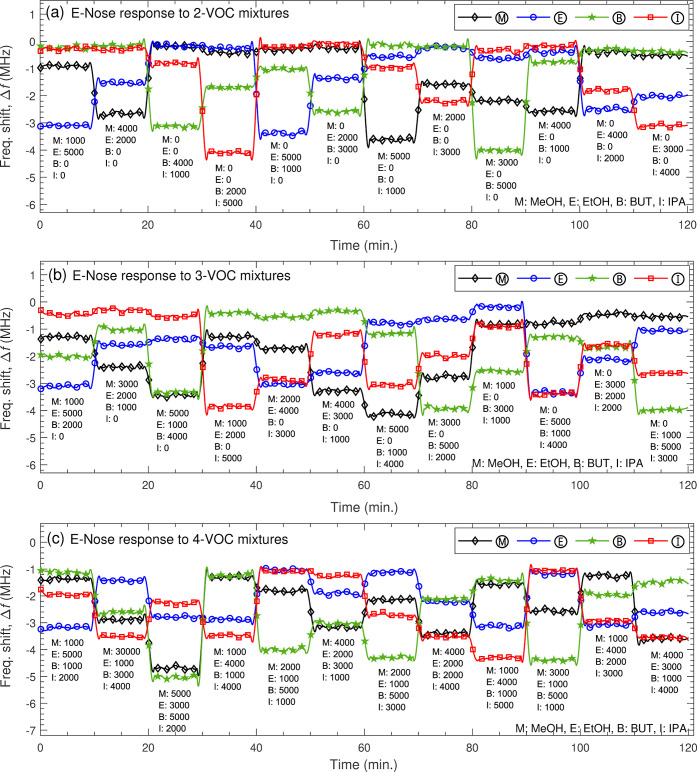
E-Nose response to mixtures of (a) two, (b) three, and (c) four VOCs at difference concentrations from 1000 to 5000 ppm.

For example, Ⓜ and Ⓔ recorded Δ*f* of 0.877 and 3.21 MHz, when exposed to a mixture of MeOH (1000 ppm) and EtOH (5000 ppm), respectively. However, these values were 0.669 and 3.15 MHz when measured independently (see Figure 8). Similarly, Ⓑ measured Δ*f* = 1.006 MHz for a mixture of BUT (1000 ppm) and EtOH (5000 ppm), while it recorded Δ*f* = 0.848 MHz when exposed to BUT alone. The effect of cross-sensitivity increased further with three gases and became severe with four, as shown in Figure 11b,c. For example, Ⓜ detected Δ*f* = 1.26 MHz to 1000 ppm of MeOH when mixed with two VOCs—EtOH (5000 ppm) and BUT (2000 ppm)—and Δ*f* = 1.37 MHz when combined with a mixture of three VOCs—EtOH (5000 ppm), BUT (1000 ppm), and IPA (2000 ppm). A single-sensor system would misinterpret these responses as 1883 ppm and 2047 ppm instead of the actual 1000 ppm concentration.

For example, Ⓜ and Ⓔ recorded Δ*f* values of 0.877 and 3.21 MHz for a MeOH (1000 ppm) and EtOH (5000 ppm) mixture, respectively. When measured independently, these values were 0.669 and 3.15 MHz (see Figure 8). Similarly, Ⓑ measured Δ*f* = 1.006 MHz for a BUT (1000 ppm) and EtOH (5000 ppm) mixture, compared to 0.848 MHz for BUT alone. The effect of cross-sensitivity increased with three or more gases, as shown in Figure 11b,c. For example, Ⓜ recorded Δ*f* = 1.26 MHz for a mixture of MeOH (1000 ppm), EtOH (5000 ppm), and BUT (2000 ppm), and Δ*f* = 1.37 MHz with an additional VOC—IPA (2000 ppm). A single-sensor system would also misinterpret these as 1883 ppm and 2047 ppm, rather than the actual 1000 ppm.

Without a mitigation mechanism, sensors can reliably detect a target VOC only in isolation. However, in real-world environments, it is often unclear whether the sensor is measuring the target alone or within a complex multi-VOC mixture. Accurately detecting and quantifying multiple VOCs in such mixtures is challenging, even with highly selective sensors, leading to potential misidentification or quantification errors. The impact of mixture complexity on measurement accuracy highlights the limitations of traditional single sensors. However, cross-reactivity patterns, extracted with minimal EM influence, suggest that E-Nose could improve prediction accuracy and reduce errors in multi-VOC detection and concentration estimation through advanced ML algorithms.

### Data Set Analysis

The correlation matrix (Figure 12) illustrates the relationships between sensor responses and VOC concentrations using a color-coded heatmap that intuitively conveys both the strength and direction of each correlation. Frequency responses show strong correlations with their respective target gases, highlighting each sensor’s primary response to specific VOCs. For instance, Δ*f*_1_ correlates strongly with *C*_MeOH_, while Δ*f*_2_, Δ*f*_3_, and Δ*f*_4_ are primarily associated with *C*_EtOH_, *C*_BUT_, and *C*_IPA_, respectively. However, these correlations are not entirely precise, reflecting the complexity of sensor behavior across varying VOC concentrations, likely due to nonlinearities or overlapping influences shaping response patterns. Off-diagonal correlations in frequency responses reveal moderate cross-reactive signals from other sensors. These signals are critical for VOC concentration estimation and provide insights into sensor behavior under mixed exposures. In contrast, magnitude responses (Δ*S*_11_ to Δ*S*_44_) exhibit a more selective pattern, with strong correlations limited to specific cases. For example, Δ*S*_11_ shows a notable secondary correlation with *C*_MeOH_, while Δ*S*_22_, Δ*S*_33_, and Δ*S*_44_ demonstrate selective dominance with *C*_EtOH_, *C*_BUT_, and *C*_IPA_, respectively. Other magnitude responses exhibit weaker correlations, indicating limited or noisier contributions. This selective sparsity ensures that magnitude responses primarily capture dominant nonlinear patterns, with cross-reactive signals playing a secondary role.

**Figure 12:**
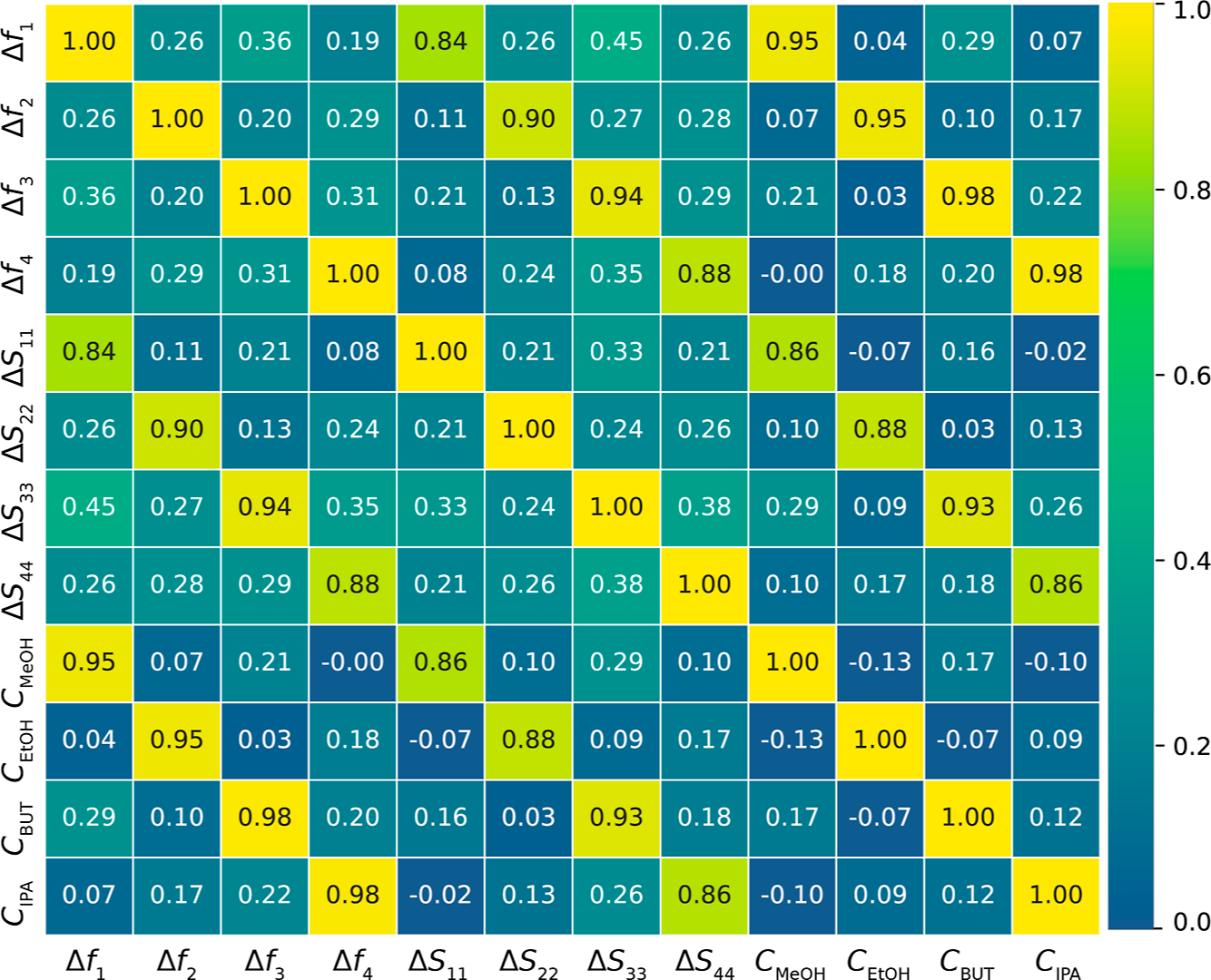
Correlation matrix showing relationships between sensor responses and VOC concentrations, with strong primary responses along the diagonal and moderate cross-reactivity off-diagonal.

### Neural Network for Concentration Estimation

The model performance was evaluated on the evaluation data set **E**, with *R* ^2^ values of 0.991, 0.985, 0.990, and 0.982 for MeOH, EtOH, BUT, and IPA, respectively, demonstrating its strong robustness and predictive accuracy. Figure 13 compares the actual (“○”) and predicted (“×”) concentrations for 100 randomly selected samples from **E**, with panels (a–d) representing MeOH, EtOH, BUT, and IPA, respectively. The close agreement between predicted and actual values, as indicated by their alignment (“⊗”) across most samples, demonstrates the model’s effectiveness. Minor discrepancies at certain concentrations suggest minimal prediction errors. This analysis confirms the model’s capability to capture concentration patterns with high precision. While the predicted concentrations closely follow the actual values, as shown by the clustering around the diagonal, slight deviations are observed at specific concentrations, as highlighted by the error bars in Figure 14a–d, which illustrate the absolute prediction errors for each VOC.

**Figure 13:**
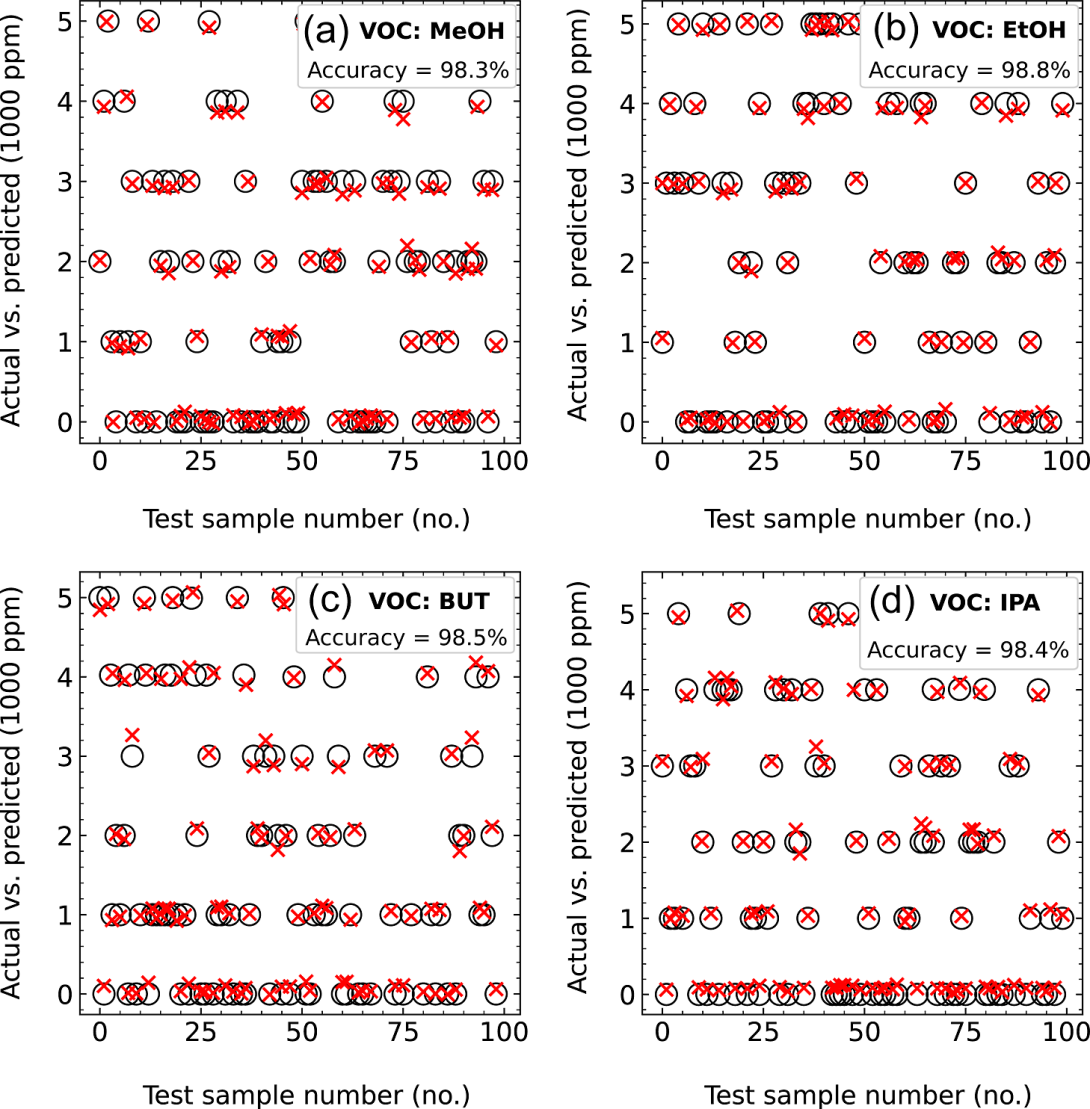
Actual vs predicted VOC concentrations for (a) MeOH, (b) EtOH, (c) BUT, and (d) IPA, evaluated on 100 random test samples. Actual and predicted values are marked by “○” and “×”, respectively, demonstrating the model’s precision and reliability.

**Figure 14:**
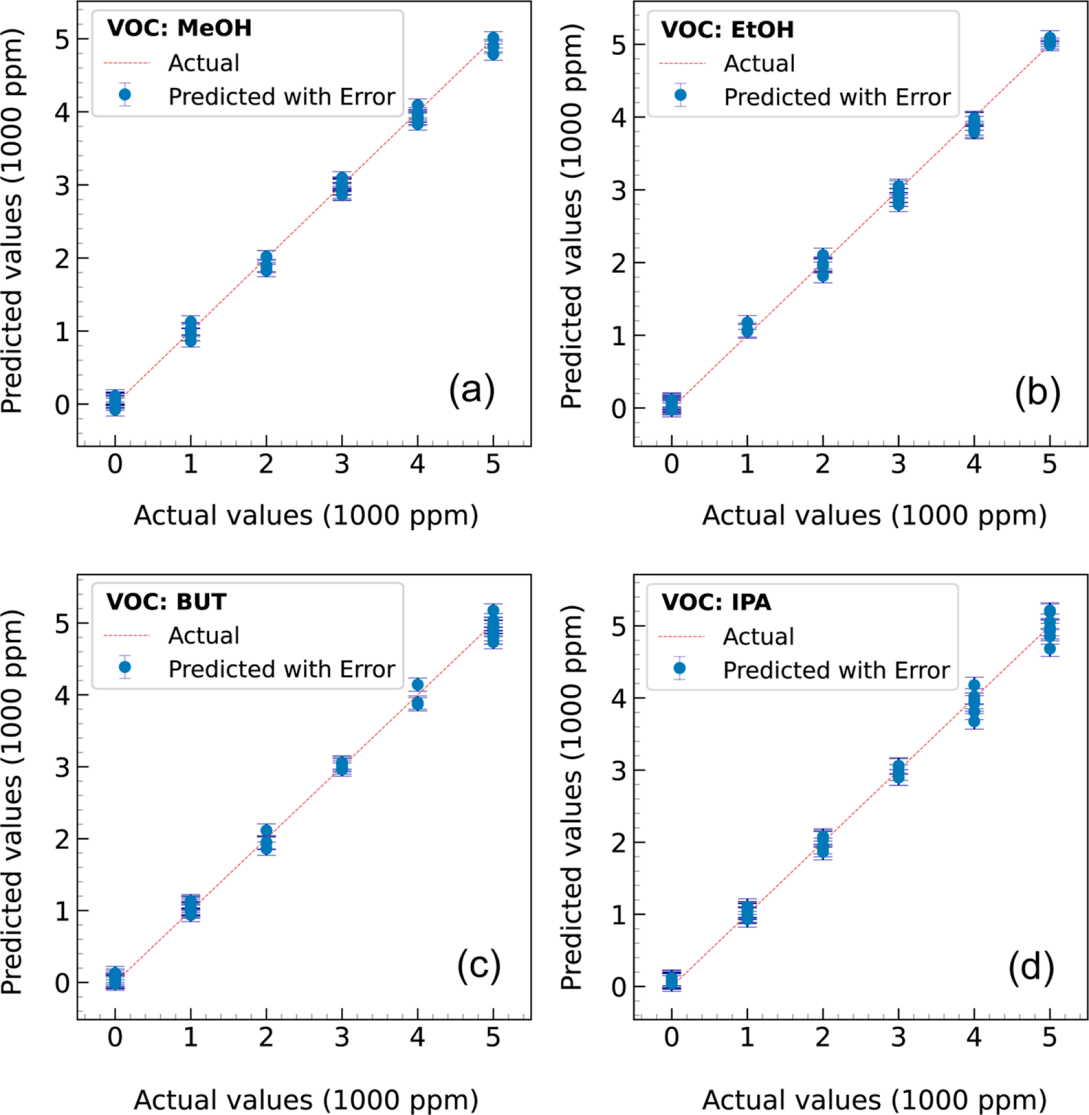
Prediction errors with error bars for (a) MeOH, (b) EtOH, (c) BUT, and (d) IPA, indicating slight deviations at different concentrations.

The sensing elements exhibited varying cross-reactivity to VOCs, influenced by their concentrations and mixture complexities, creating a nonlinear relationship within the sensor array responses. Stronger interference at higher concentrations suggests this effect could enhance E-Nose predictions. Figure 15 illustrates the distribution of average prediction errors across VOC types, concentrations, and mixture complexities, highlighting their effects on prediction accuracy.

**Figure 15:**
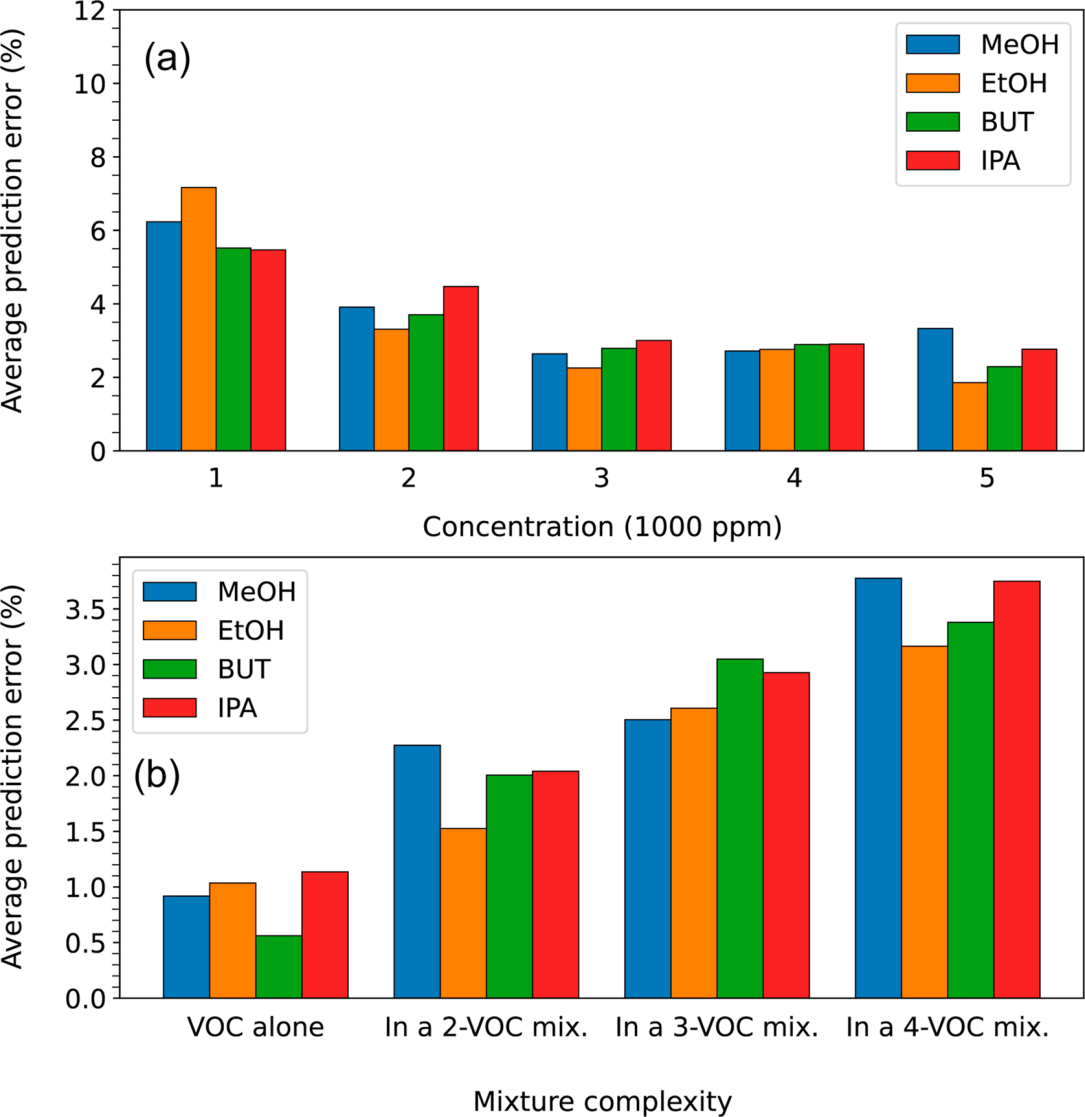
Breakdown of prediction error by VOC type, concentration, and mixture complexity. (a) Average prediction error as a function of concentration, showing improved accuracy at higher concentrations. (b) Effect of mixture complexity on prediction error, with minimal error for simple mixtures and increasing error as mixture complexity rises.

As the concentration increases, the average prediction error decreases consistently across all VOCs, indicating E-Nose’s improved prediction accuracy (∼99%) at higher concentrations (see Figure 15a). However, the prediction errors are noticeably higher than average at lower concentrations. Figure 15b illustrates the effect of mixture complexity on prediction error. Model showed almost perfect estimation (with ∼0.25% error) when VOCs were measured alone, while as mixture complexity increases, the average prediction error rises (up to 1.50%) for all four VOCs.

The E-Nose demonstrates varying degrees of cross-reactivity to different VOCs, influenced by their concentrations, resulting in a complex, nonlinear relationship (see Section Decoupled E-Nose Response to Single VOCs). At higher concentrations, interference from cross-reactive sensors becomes more pronounced but can be exploited to improve the E-Nose’s predictive accuracy. Figure 15 illustrates prediction error distributions across VOC types, concentrations, and mixture complexities. In Figure 15a, average prediction error is plotted against VOC concentration, revealing a decrease in error as concentration increases. This highlights the E-Nose’s improved accuracy, rising from 95% at lower concentrations to 98% at higher levels. Figure 15b explores the influence of mixture complexity on prediction error. The model achieves near-perfect accuracy with an error of 0.5% for individual VOCs. As the mixture complexity increases (mixtures containing two to four VOCs), the average prediction error rises to 2.0% to 3.5%.

Table 3 presents a comprehensive comparison of recent advanced E-Noses from the literature with the proposed microwave MIMO E-Nose, highlighting its key features. In addition to multigas sensing, the proposed E-Nose ensures uninterrupted communication—a feature missing in traditional CR-based sensors. With MIMO technology utilizing four antenna elements to deliver high data rates, the system addresses the demands of next-generation wireless communication. Wireless devices powered by this cutting-edge E-Nose enable high-speed data connectivity, real-time AQ monitoring, and seamless integration with WSNs, paving the way for smarter and more efficient environmental monitoring.

**Table 3:** Proposed E-Nose Sensor Features Compared with Recent Advances^a^

	ref 13	ref 17	ref 18	ref 19	ref 20	ref 21	this work
sensor type	commercial CR	CR-MOS	MEMS-MOS	CR-MOS	CR-MOS	CR-MOS	antenna + MIP
no. of sensors	8	8	6	2	1	1	4
no. of target gas	12	6	6	5	5	4	4
operating conditions	N/A	250 °C	200 °C	UV activation	RT	280 °C	RT
ML method	LSTM-CNN	CNN	voting method	CNN	CNN	LDL	NN
accuracy (%)	95.31	98.1	96.8	99.32	93.9	95	95–98
error (%)	3.9	10.15	∼1	13.82	19.8	11.9	2–3.5
WSN suitability	no	no	yes	no	no	no	yes
operation	sensor	sensor	sensor	sensor	sensor	sensor	dual-functional

a CNN: convolutional neural network, LDL: lightweight deep learning, LSTM: long short-term memory.

The developed solution effectively detects VOC mixtures and estimates concentrations in indoor environments while also extending to outdoor AQ monitoring, where complex gas mixtures are prevalent. This versatility enables comprehensive AQ assessments, addressing critical gaps in indoor and outdoor air quality management.

Our results demonstrate the potential of the presented proof-of-concept dual-functional sensor system for practical applications. The next logical step is to evaluate the sensor’s performance under real-world conditions–such as varying humidity levels (30–90% RH) and temperatures (0–40 °C)–to comprehensively assess its robustness and stability. Future work will also include a comparative analysis with state-of-the-art microwave gas sensors to further establish its applicability and commercial readiness. Although antenna stability during sensing was confirmed, a complete evaluation of communication metrics will be conducted in subsequent studies to fully demonstrate seamless dual-function operation.

## Conclusions

This research presents a significant advancement in AQ monitoring by combining precision sensing with scalable deployment, providing a transformative solution to critical environmental, medical, industrial, and economic challenges. We introduce the first dual-functional, antenna-based microwave MIMO E-Nose, capable of selective VOC detection, high-precision concentration estimation, and uninterrupted wireless communication, seamlessly integrating into WSNs for IoT applications. The E-Nose utilizes a 4-port MIMO antenna optimized with MIP/MWCNT-based materials for exceptional analyte recognition, coupled with a dual-branch neural network (NN) that mitigates cross-sensitivities and addresses VOC mixture complexities. This combination enables highly sensitive detection, meeting safety thresholds for clean air standards across environmental, industrial, and healthcare applications. Addressing novel challenges such as cross-reactivity with EM interference, optimizing sensing structures and sensor positioning, and employing domain-aware ML techniques enables the model to achieve high predictive accuracy (*R* ^2^ = 0.982 to 0.991) with minimal prediction errors for pure VOCs (0.5%) and mixtures (2 to 3.5%). The E-Nose furthermore delivers robust antenna performance, including a 511 MHz operating bandwidth, 15 dB isolation bandwidth, 81.5% efficiency, support for the high data rate demands of 5G/6G and next-generation wireless communication services, and stable radiation patterns, ensuring coverage and seamless integration into WSNs. Its low-cost fabrication, compact design (2.5 × 12.2 cm), and scalable architecture enable large-scale deployment, bridging the gap between reliable sensing and practical implementation. Its customizable design paves the way for detecting a wider range of VOCs and toxic gases, advancing efforts to mitigate air pollution’s health impacts and supporting global sustainability goals.

## Supplementary Material



## Notes

SThe Supporting Information is available free of charge at https://pubs.acs.org/doi/10.1021/acssensors.5c00243.

This document provides supplementary details on the FT-IR analysis of MeOH-selective MIP films, optimization of the sensing structure and hyperparameters for VOC prediction, experimental setup for sensing elements, performance analysis of sensing components, decoupling and matching techniques, coupled sensor response, radiation pattern measurements, and polynomial fit and calibration curves for VOC detection (PDF)
