# Supplementary material for: Microwave MIMO E‑Nose for Wireless Communication and Selective Detection of VOC Mixtures with Concentration Estimation
Source: ACS Sens. 2025 Aug 25;10(9):6446–63. doi: 10.1021/acssensors.5c00243 (PMC12481560; doi:10.1021/acssensors.5c00243)
Supplement: Supplementary file 1 [file se5c00243_si_001.pdf]

(Supporting Information)

Microwave MIMO E-Nose for Wireless Communication and Selective Detection of VOC Mixtures with Concentration Estimation

Mohammad Mahmudul Hasan,[*,†] Onur Alev,[†,‡] Pavel Skrabanek,[¶] Gabriela Soukupová,[§] Fatima Hassouna,[§] and Michael Cheffena[†]

†*Faculty of Engineering, Norwegian University of Science and Technology (NTNU), Gjøvik 2815, Norway*

‡*Department of Physics, Gebze Technical University, 41400 Gebze, Kocaeli, Turkey*

¶*Faculty of Mechanical Engineering, Brno University of Technology, Brno 61200, Czech Republic*

§*Faculty of Chemical Engineering, University of Chemistry and Technology, Prague 16628, Czech Republic*

E-mail: mohammad.m.hasan@ntnu.no

1 FT-IR Analysis of MeOH-Selective MIP Film

FT-IR spectra were recorded using a PerkinElmer Spectrum 3 spectrometer in the range of 600–4000 cm^{-1}. For the measurements, the synthesized MeOH-selective MIP was coated onto glass substrates using the same amount and drop-casting method as applied on the antenna transducer. Prior to coating, the glass substrates were cleaned sequentially with acetone, ethanol, and deionized water following a standard cleaning procedure. The FT-IR results are presented in Figure S1 below.

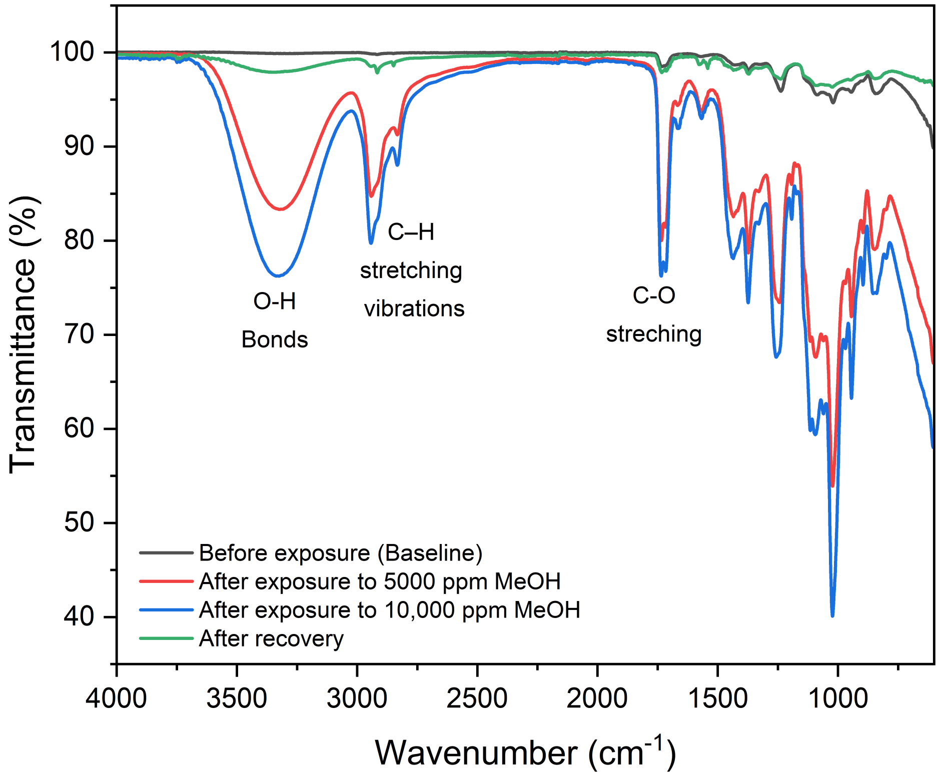

Figure S1: FT-IR spectra of the MeOH-selective MIP film at different concentrations.

2 Optimization of the sensing structure

The sensing structures were optimized based on material properties, including conductivity ($\sim 2.1 \times 10^{-3}$ S), impedance ($480\,\Omega$ - j$72.5\,\Omega$), and dielectric constant (136), measured with an LCR bridge meter (R&S HM8118). The structure was modeled in COMSOL Multiphysics with the sensing material placed between the fingers. The simulation of the electric field distribution with a DC voltage of 1.0 V across the IDE contacts showed maximum field strength at the electrode edges, decreasing toward the gap centers.

3 Optimizing Hyperparameters for VOC Prediction

The hyperparameters of the Dual-Branch Neural Network for E-Nose include the activation function $\phi(\cdot)$, the regularization strengths α_1 and α_2, the dropout probability $p^{(3)}$, and the number of neurons $N_f^{(i)}$, $N_m^{(i)}$ and $N^{(3)}$, where $i \in \{1, 2\}$. Here, f and m denote the frequency and magnitude branches, respectively. Furthermore, the learning rate η, the number of epochs N_ε, and the batch size S_b are also considered hyperparameters.

A grid search, combined with stratified k-fold cross-validation (5 splits, 15 repetitions), was employed to identify the optimal hyperparameter settings. The search space included $N_f^{(1)}, N_f^{(2)}, N_m^{(1)}, N_m^{(2)}, N^{(3)} \in \{16, 32, \ldots, 256\}$, $\alpha_1, \alpha_2 \in \{0.001, 0.005, \ldots, 0.5\}$, $p^{(3)} \in \{0.1, 0.2, \ldots, 0.5\}$, $\phi(\cdot) \in \{\tanh, \mathrm{ReLU}, \text{leaky ReLU}\}$, $\eta \in \{0.001, 0.005, \ldots, 0.1\}$, $S_b \in \{16, 32, \ldots, 64\}$, and $N_\varepsilon \in \{10, 20, \ldots, 500\}$. Training was terminated early if no improvement in validation performance was observed for 10 consecutive epochs.

Model performance was evaluated using MSE, with the lowest average MSE across cross-validation determining the best hyperparameter configuration. The hyperparameter search was performed on the training dataset $\mathbf{T}$. The best configuration included $N_f^{(1)} = 64$, $N_f^{(2)} = 32$, $N_m^{(1)} = 32$, $N_m^{(2)} = 16$, $N^{(3)} = 64$, $\alpha_1 = 0.02$, $\alpha_2 = 0.01$, $p^{(3)} = 0.2$, $\phi(\cdot) = \mathrm{ReLU}$, $\eta = 0.01$, $S_b = 32$, and $N_\varepsilon = 50$.

4 Experimental setup for the sensing elements

For CR sensing, the functionalized IDE elements were mounted on a PCB (custom-made) in an array configuration and placed in a 0.24 L airtight chamber. An Arduino-based switching circuit sequentially connected each IDE element to an LCR bridge meter using a 4-wire measurement system with a sampling interval T_s =1.25 s, ensuring uniform data collection across all sensing elements. Stable baseline resistances R_a were recorded first for each sensing element. Precise amounts of liquid VOCs were then evaporated into the chamber using a precision micro-pipette, with quantities determined based on the ideal gas law. Upon VOC exposure, dynamic changes were observed, and both response time τ_{res} and recovery time τ_{rec} were recorded. Once the sensors reached equilibrium, stable responses R_g from all IDE elements were recorded using LabVIEW software interfaced with the DMM. The sensor response was calculated as $\Delta R = (R_g - R_a)/R_a$. For recovery, the chamber lid was opened, allowing the system to return to its initial baseline resistance.

5 Performance analysis of the sensing components

The R_a values for IDE/MeOH, IDE/EtOH, IDE/BUT, and IDE/IPA were 440 Ω, 385 Ω, 490 Ω, and 310 Ω, respectively. When exposed to their target VOCs at 1000 to 10 000 ppm, the sensors exhibited rapid, proportional responses, confirming linear detection within $\tau_{res} =$ 3.85 to 6.5 min, as shown in Fig. S2 (Supporting Information). Upon gas removal, desorption of molecules restored sensor resistance to baseline, confirming reversibility within $\tau_{rec} =$ 1.1 to 2.3 min. Additionally, the sensing elements showed strong selectivity for their target

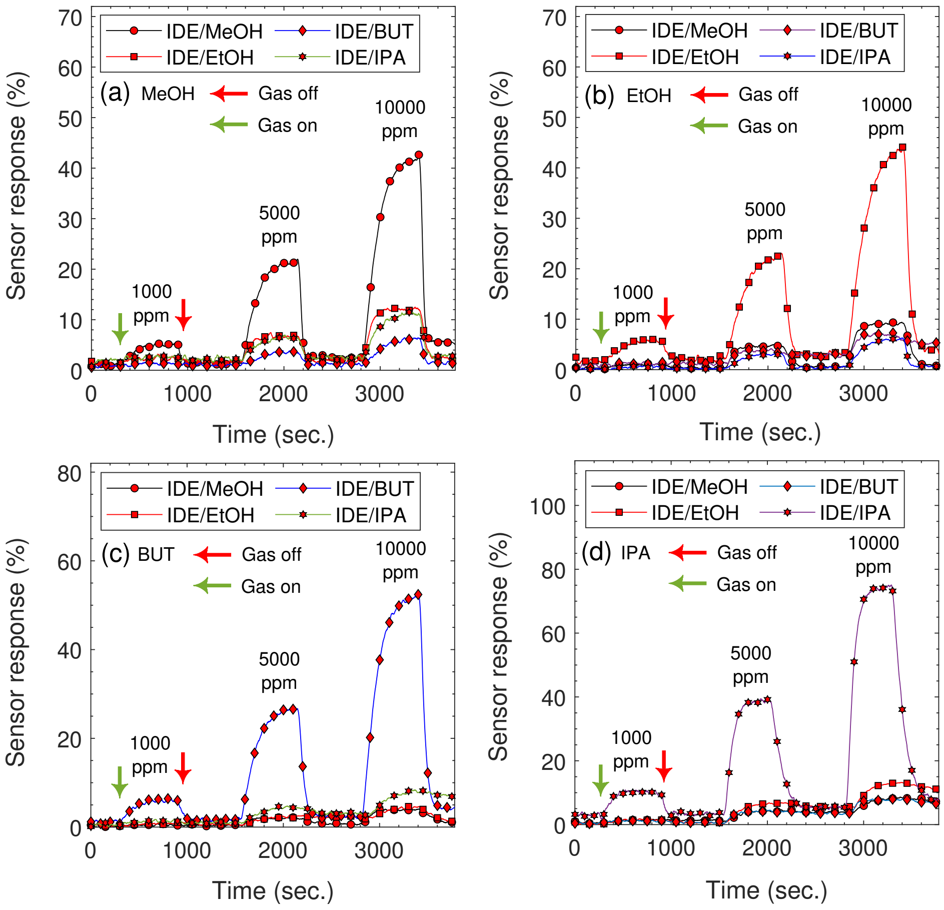

Figure S2: Response of individual sensing elements to (a) MeOH, (b) EtOH, (c) BUT, and (d) IPA at 1000 to 10 000 ppm, showing selectivity and cross-reactivity.

VOCs.

Several issues were evident across all sensing elements. First, baseline shifts of approximately 5 to 9 % were observed after the recovery phase, potentially causing residual or incremental responses if the sensors did not fully recover between exposures. The severity of these shifts increased non-linearly with concentration and varied across VOCs, a common phenomenon in CR gas sensing at RT.[1] Second, cross-sensitivity interference was noticeable, potentially leading to false readings. For instance, the IDE/MeOH sensor exhibited a strong selectivity with $\Delta R = 41\,\%$ for 10 000 ppm of MeOH, while cross-sensitivities for other VOCs ranged from 6 to 11 %. These issues, including baseline shifts and cross-sensitivity, were observed across all sensing components and are likely to affect the E-Nose system if not mitigated. Thirdly, the primary and secondary cross-reactive compounds for each sensing element were identified.

6 Decoupling and Matching Techniques

The E-Nose is modeled as a π-network, characterized by scattering S_{ij} and admittance Y_{ij} parameters, where i and j represent the port numbers. Due to the symmetric design, a

two-element antenna array was used for simplicity, with mutual admittance Y_{21} representing the coupling between the ports. To achieve port isolation, the real part of the admittance $\mathfrak{Re}\{Y_{21}\}$ must be zero, ensuring that Y_{21} is purely imaginary. This ensures that the coupling is reactive and can, therefore, be neutralized with a reactive component (inductive or capacitive).[2] Transmission lines (T-lines) were incorporated into the antenna feeds to adjust S-parameter phases and modify the antennas' total admittance. The desired phase shifts φ_t can be achieved using T-lines of appropriate lengths l_t to transform Y_{21} into a purely imaginary component $\mathfrak{Im}\{Y_{21}\}$.[2,3] The element value X_P is determined by $X_P = 1/\mathfrak{Im}\{Y_{21}\}$, with $\mathfrak{Re}\{Y_{21}\} \approx 0$.

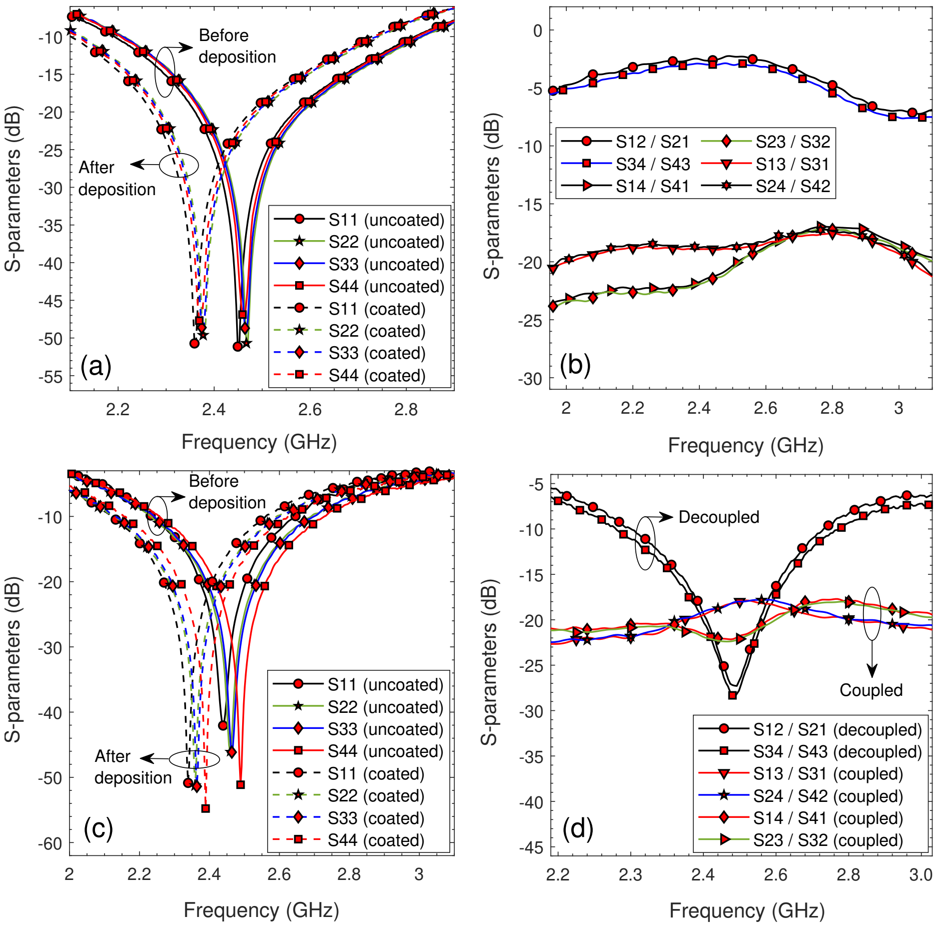

Figure S3: Measured reflection (S_{ii}) and transmission (S_{ij}) coefficients before and after coating with sensing material for (a)-(b) coupled and (c)-(d) decoupled configurations.

The reference planes t_1, t_2, and t_3 correspond to the stages of antennas alone, antennas with T-lines, and antennas with T-lines, decoupling, and matching circuits, respectively. The S-parameters at t_1 were first obtained using an EM simulator (HFSS). Then, suitable l_t values were identified using a circuit simulator (Keysight ADS) to achieve the desired φ_t at the input ports. While multiple l_t values can satisfy the phase conditions for decoupling,[2,3] it is essential to select values compatible with commercially available lumped elements to ensure practical implementation. The reactive component changes the antenna system's total reactance, shifting its resonance frequency. To restore resonance, a matching network (MN)

compensates for the reactance changes. Two identical L-section MNs were employed due to the symmetric antenna elements, with reactive components ($X_C = C_1, C_2$ and $X_L = L_1, L_2$). The component values were $C_1, C_2 = 0.95\,\mathrm{pF}$, $C = 1.00\,\mathrm{pF}$, and $L_1, L_2 = 3.3\,\mathrm{nH}$. The l_t values were optimized using S-parameter models of the lumped elements provided by the manufacturer, and a 24.76 mm T-line was added at the antenna feed points. Finally, the DN and MN circuits were integrated into the T-lines.

7 Coupled Sensor Response

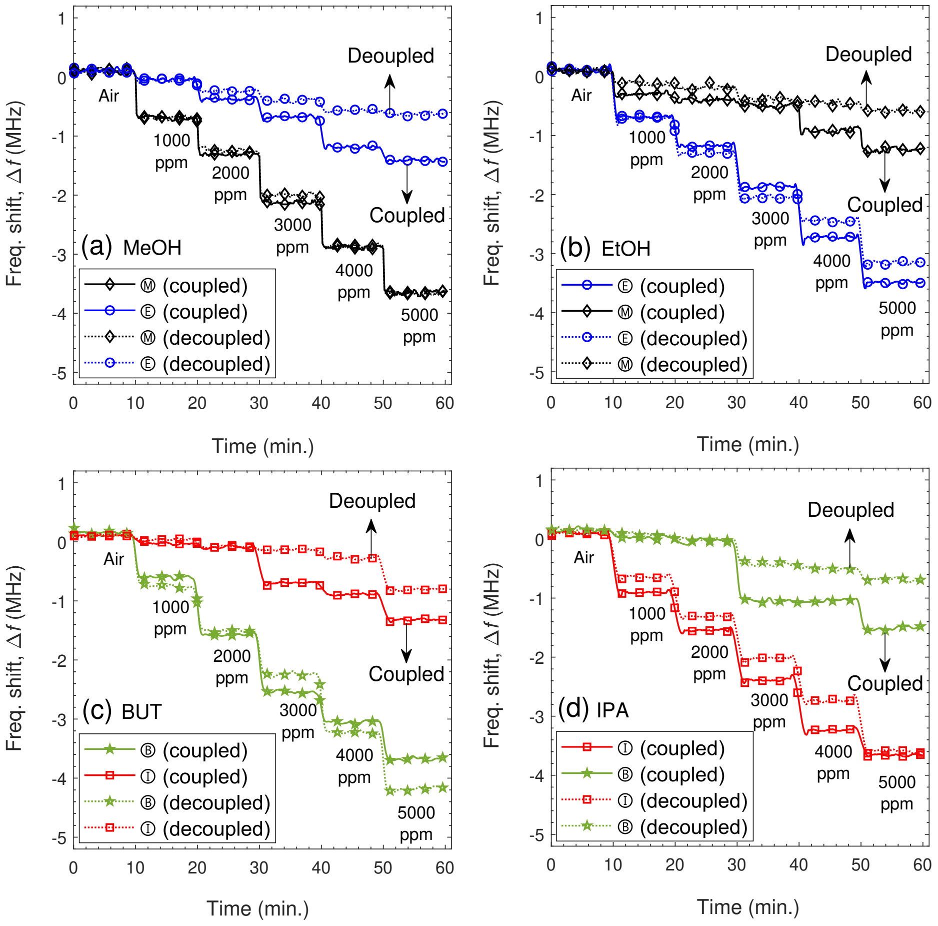

Figure S4: E-Nose response (Δf) during gas exposure for (a) MeOH, (b) EtOH, (c) BUT, and (d) IPA at 1000 to 5000 ppm, illustrating the combined effects of cross-sensitivity and mutual coupling interference on gas sensing.

8 Radiation Pattern

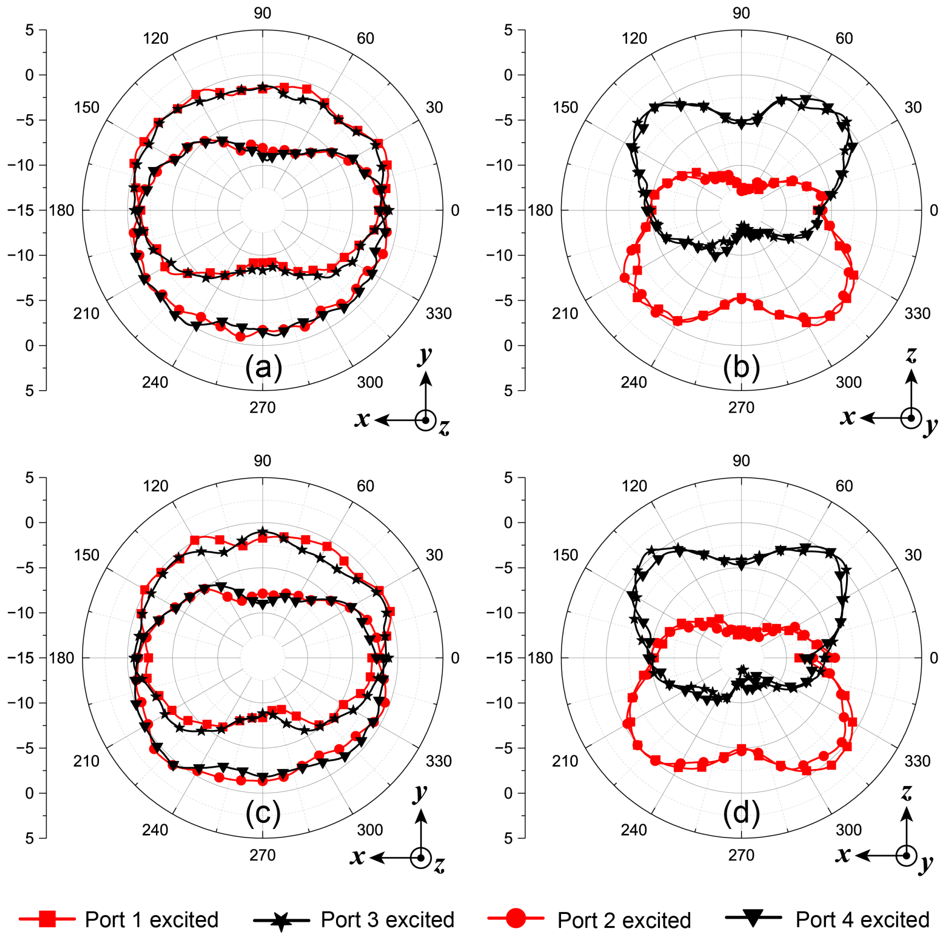

Figure S5: Measured E(yz)-plane (left) and H(xz)-plane (right) of the E-Nose: (a)-(b) before and (c)-(d) after deposition of the sensing material.

9 Polynomial Fit and Calibration Curves

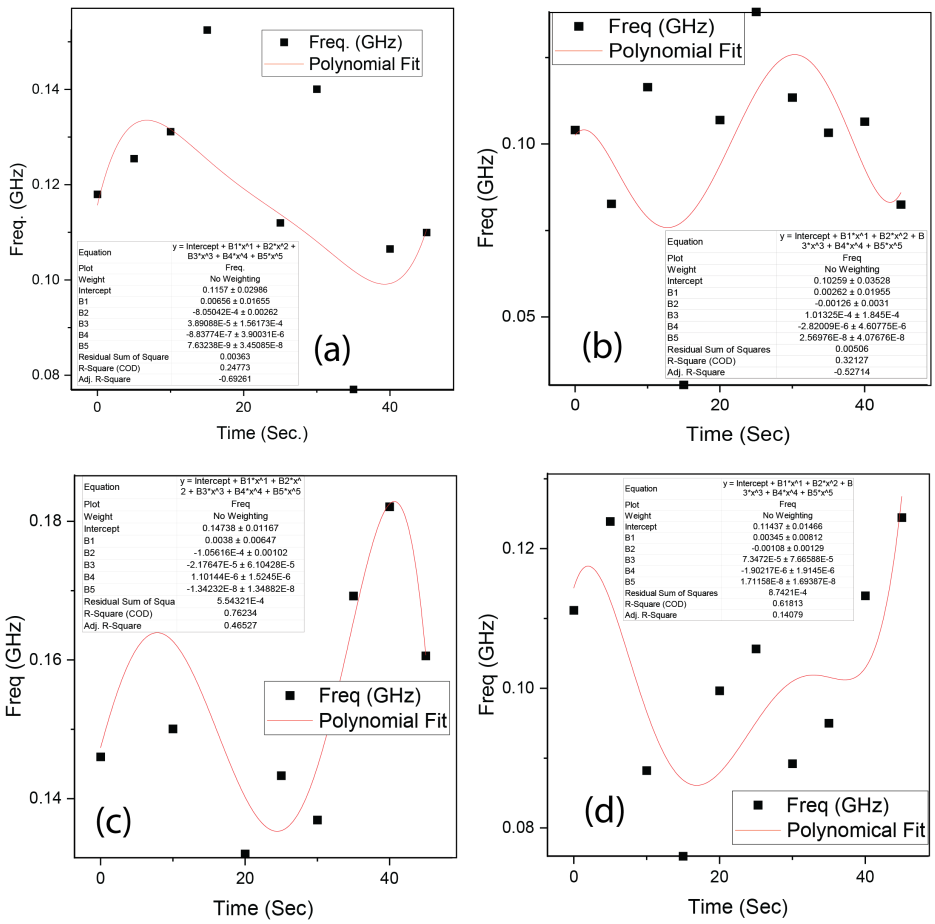

Figure S6: Polynomial fit for (a) MeOH, (b) EtOH, (c) BUT, and (d) IPA.

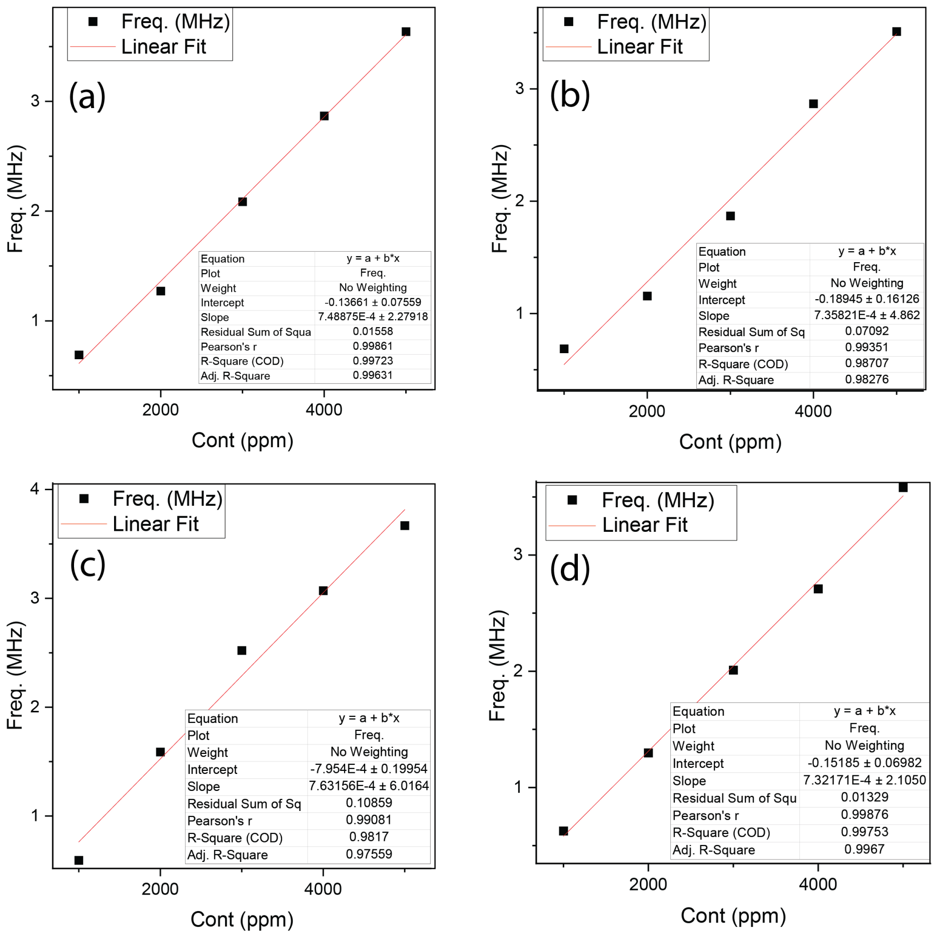

Figure S7: Calibration curves for (a) MeOH, (b) EtOH, (c) BUT, and (d) IPA.

References

(1) Srinivasan, P.; Ezhilan, M.; Kulandaisamy, A. J.; Babu, K. J.; Rayappan, J. B. B. Room temperature chemiresistive gas sensors: challenges and strategies—a mini review. *Journal of Materials Science: Materials in Electronics* **2019**, *30*, 15825–15847.

(2) Chen, S. C.; Wang, Y. S.; Chung, S. J. A decoupling technique for increasing the port isolation between two strongly coupled antennas. *IEEE Transactions on Antennas and Propagation* **2008**, *56*, 3650–3658.

(3) Venkatasubramanian, S. N.; Li, L.; Lehtovuori, A.; Icheln, C.; Haneda, K. Impact of using resistive elements for wideband isolation improvement. *IEEE Transactions on Antennas and Propagation* **2017**, *65*, 52–62.
